# Uncovering shared enzymes and secondary routes across β-oxidation cycles in *Mycolicibacterial* phytosterol side-chain degradation

**DOI:** 10.1186/s12934-026-03087-7

**Published:** 2026-08-12

**Authors:** Qianqian Ma, Jianxin He, Yupan Zhou, Xirenayi Tuergong, Ye Wang, Jiaxue Chang, Jiayi Wang, Alimire Ruzi, Xiyao Cheng, Shikui Song, Zhengding Su

**Affiliations:** 1 School of Pharmaceutical Sciences, Institute of Materia Medica, Xinjiang University, Urumqi, 830017 Xinjiang China; 2 Laboratory of Protein Engineering and Biopharmaceutical Sciences, Hubei University of Technology, Wuhan, 430068 China; 3 School of Light Industry and Food Engineering, Guangxi University, Nanning, 530004 Guangxi China; 4 Xinjiang Key Laboratory of Special Species Conservation and Regulatory Biology, College of Life Sciences, Xinjiang Normal University, Urumqi, 830054 Xinjiang China

**Keywords:** Phytosterols, Bioconversion, Metabolic pathway, *Mycolicibacterium neoaurum*, Androstane

## Abstract

*Mycolicibacterium* strains are among the most effective biofactories for converting phytosterols into active pharmaceutical intermediates. Despite the main route resembling fatty acid β-oxidation, the specific enzymes and their precise roles are poorly defined. In this study, we performed a comprehensive gene knock-out analysis in the industrial 4-AD-producing strain, *Mycolicibacterium neoaurum* HGMS9 strain, a variant of *M. neoaurum* B-3805. Our results demonstrate that the three β-oxidation cycles responsible for phytosterol side-chain degradation share a suite of core enzymes. Specifically, seven enzymes, including the acyl-CoA dehydrogenase ChsE1/E2, ChsE4/E5, the hydratase, ChsH1/H2 and the aldolase Ltp2, function in both the second and third β-oxidation cycles to generate the final products, 4-AD. Importantly, we identified five potential secondary routes that divert metabolism to produce eight off-route C22, C23 and C24 intermediates. These compounds represent valuable precursors for the synthesizing advanced steroids like corticosteroids and bile acids. This study not only clarifies the enzymatic steps of phytosterol side-chain degradation but also establishes a metabolic blueprint for engineering high-yielding *Mycolicibacterium* strains.

**Supplementary Information:**

The online version contains supplementary material available at https://doi.org/10.1186/s12934-026-03087-7.

## Introduction

Steroid compounds with a tetracyclic hydrocarbon ring structure and various functional groups find widespread clinical application [1, 2]. The synthesis of medicinal steroids heavily relies on two key starting materials: 4-androstene-3,17-dione (4-AD) and 21-hydroxy-20-methylpregn-4-en-3-one (Bisnoralcohol, BA) [3, 4]. While the genera like *Gordonia*, *Nocardia*, *Rhodococcus* and *Mycobacteria* have the capability to degrade the side-chains of cholesterol, cholic acid and phytosterols [5–7], only mycobacteria, notably *Mycolicibacterium neoaurum*, can efficiently accumulate androstane steroids, preserving their crucial tetracyclic hydrocarbon ring structures [3, 4, 8–11]. Currently, the method for obtaining these starting materials involves utilizing mycolicibacterial steroid degradation to remove the phytosterol side-chains [4, 6, 12]. *M. neoaurum* NRRL B-3805 and its parent strain NRRL B-3683 were the first microorganisms identified as suitable for the industrial production of 4-AD through natural accumulation [13]. Their metabolic activity, however, also results in the co-production of several intermediates, including small quantities of 1,4-androstadienedione (ADD), 9α-hydroxy-4-androstenedione (9OH-AD), 21-hydroxy-20-methyloregn-4-en-3-one (BA) and trace of other intermediates. Following in the footsteps of the B-3805 strain, *M. neoaurum* HGMS2 has also been employed for industrial application for nearly a decade [14]. Strain engineering of *M. neoaurum* HGMS2 has yielded specialized mutants with enhanced production yields for the target compounds 4-AD [15] and BA [16].

Bacterial steroid degradation encompasses three distinct pathways including cholesterol, cholate and testosterone degradation pathways [4, 17, 18]. Genome analysis has unveiled that these pathways share a core mechanism of 9,10-secosteroid intermediate [17, 19]. The availability of genomic data for both pathogenic *M. tuberculosis* strains and the nonpathogenic model *M. smegmatis* MC^2^ 155 has provided insights into the study of the phytosterol side-chain degradation pathway in 4-AD-producing strains [20, 21]. Enzymatic and genomic investigations of 4-AD-producing *M. neoaurum* strains, including NRRL B-3805, NRRL B-3683, HGMS2 and others, have revealed that their phytosterol side-chain degradation pathway involves at least 16 enzymes acting in 13 consecutive steps to produce 4-AD [4, 6, 14, 17, 22–26].

During the microbial production of 4-AD, several intermediates are generated [27, 28]. Although these intermediates pose serious challenges for the 4-AD purification, they can serve as valuable precursors for the synthesis of other steroid medicines. Gene knockout studies reveal that different mycobacterium neoaurum accumulate distinct intermediates due to variations in their metabolic routes. Knocking out the genes for 3-ketosteroid-^Δ^1-dehydrogenase and 3-ketosteroid 9α-hydroxylases in *M. neoaurum* HGMS2 created a mutant that efficiently produces 4-AD, albeit with minor accumulation of small amount [15, 16]. Other mutants accumulate specific precursor with pharmaceutical potential: 3-oxochol-4-en-24-carboxylic acid (3-OCO) for bile acid drug [29], and 3-oxo-4,17 (20)-pregnadiene-20-carboxylic acid (3-OPDC) for hydroxyprogesterone [30]. In *M. neoaurum* CCTCC AB2019054, deleting acyl-CoA dehydrogenase and acyl-CoA hydratase genes, *chsE1/E2* and *chsH1/chsH2*, promotes the accumulation of 3-oxo-4-pregnadiene-20-carboxylic methyl ester (3-OPCM) and 3-oxo-4,17-pregnadiene-20-carboxylic methyl ester (3-OPDCM) [27]. In *M. neoaurum* ATCC25795, knocking out the *chsH1/H2* genes leads to 3-OPDCM accumulation, and inactivating an acyl-CoA aldolase, *ltp2*, results in the inactivation of the ChsH1/H2 enzymes, while knocking out the *chsE1/E2* gene has no significant effect on the metabolism [30]. In *M. neoaurum* DSM 44,074, the knockout of the *chsE1/chsE2* and *chsE4/chsE5* genes causes the strain to accumulate 3-OPCM, and further deletion of the *chsE3* gene completely halts the degradation of phytosterols [31]. These investigations suggest that the intermediates emerge from secondary routes stemming from the main route of phytosterol side-chain degradation to accumulate 4-AD. However, our understanding in the connection of these secondary routes to the main route and related enzymes in mycolicibacterial phytosterol side-chain degradation so far remain unclear.

In this study, we would like to identify critical enzymes determining the connection between the main phytosterol side-chain degradation route and its associated secondary routes in *Mycolicibacterium neoaurum* strains. Considering the genome of the B-3805, a mutant of B-3683, is nearly identical to that of the HGMS2 strain, we were thus focused on the three β-oxidation cycles of phytosterol side-chains in the B-3805 strain. The results obtained from this work illustrate mycolicibacterial pathway of phytosterol side-chain degradation in detail, potentially facilitating the development of more efficient mycolicibacterial chassis cells and, ultimately, enhancing the production of valuable steroid intermediates.

## Materials and methods

### Reagents and biochemicals

Restriction endonucleases and *Taq* DNA polymerase were obtained from Takara (Dalian, China), while other molecular biology reagents were of the highest grade and sourced from Thermo Scientific (Shanghai, China). Phytosterol, 4-androstene-3,17-dione (4-AD) and 21-hydroxy-20-methylpregn-4-en-3-one (BA) were purchased from Sigma-Aldrich (Shanghai, China). The phytosterols obtained from the commercial source had a purity of 98% β-sitosterol, with a molecular weight of MW = 414.71 Da.

### Strains and culture

*Mycolicibacterium neoaurum* NRRL B-3805 (DSM No.: 2967) was obtained from Deutsche Sammlung von Mikroorganismen und Zellkulturen (DSMZ). Other strains utilized in this study are listed in Table 1.

**Table 1:** List of strains used in this work

Name	Description	Metabolites	Source
B3805	*Mycolicibacterium neoaurum* NRRL B-3805	4-AD, ADD	DSMZ
HGMS8	Combined deletion of *kstD211*,* kshA395*and *kshB122 *in *M. neoaurum *HGMS2	4-AD, BA	Song et al. [16]
HGMS9	Combined deletion of *kstD211*,* kshA395*and *kshB122* in B3805	4-AD, BA	This study
HGMS9^*ΔchsH2*^	*chsH2* deleted in HGMS9	3-OPDC, BA, 3-OPDCM	This study
HGMS9^*Δltp2*^	*ltp2* deleted in HGMS9	3-OPDC, BA, 3-OPDCM	This study
HGMS9^*ΔchsE1*^	*chsE1* deleted in HGMS9	4-AD, BA, 3-OPC	This study
HGMS9^*ΔchsE5*^	*chsE5* deleted in HGMS9	4-AD, BA	This study
HGMS9^*ΔchsE1/ΔchsE5*^	*chsE5* deleted in HGMS9^*ΔchsE1*^	3-OPC, 3-OPCM, BA	This study
HGMS9^*ΔfadA5*^	*fadA5* deleted in HGMS9	4-AD, BA, 3-OPD	This study
HGMS9^*ΔfadA5/Δhsd4A*^	Simultaneous deletion of *hsd4A1* and *hsd4A2* in HGMS9^*ΔfadA5*^	BA, 4-AD	This study
HGMS9^*ΔfadA5/Δhsd4A/chsH2*^	Deletion of *chsH2* in HGMS9^*ΔfadA5/Δhsd4A*^		This study
HGMS9^*ΔfadA5/Δhsd4A/chsH2/fadE30*^	Deletion of *fadE30* in HGMS9^*ΔfadA5/Δhsd4A/chsH2*^		This study
HGMS9^*ΔchsH3*^	*chsH3* deleted in HGMS9	4-AD, BA	This study
HGMS9^*ΔchsH3/ΔchsH2*^	*chsH2* deleted in HGMS9^*ΔchsH3*^	3-OPDC, BA, 3-OCDO	This study
HGMS9^*ΔchsH3/Δltp2*^	*ltp2* deleted in HGMS9^*ΔchsH3*^	3-OPDC, BA, 3-OCDO	This study
HGMS9^*Δhsd4A*^	Simultaneous deletion of *hsd4A1* and *hsd4A2* in HGMS9	BA, 4-AD	This study
HGMS9^*Δhsd4A/ΔchsH3/ΔchsH2*^	Simultaneous deletion of *chsH2* and *chsH3* in HGMS9^*Δhsd4A*^	BA, 3-OCDO	This study
HGMS9^*Δhsd4A/ΔchsH3/Δltp2*^	Simultaneous deletion of in *ltp2* and *chsH3* HGMS9^*Δhsd4A*^	BA, 3-OCDO	This study
HGMS9^*ΔechA19*^	*echA19* deleted in HGMS9	4-AD	This study
HGMS9^*ΔchsE3*^	*chsE3* deleted in HGMS9	4-AD, BA	This study
HGMS9^*Δcasc*^	*casc* deleted in HGMS9	4-AD, BA	This study
HGMS9^*ΔchsE3/ΔcasC*^	*chsE3* deleted in HGMS9^*ΔcasC*^	4-AD, BA	This study
HGMS9^*ΔchsE1/ΔchsE5/ΔchsE3*^	Simultaneous deletion of *chsE1* and *chsE5* in HGMS9^*ΔchsE3*^	3-OPC, 3-OPCM, BA	This study
HGMS9^*ΔchsE1/ΔchsE5/ΔchsE3/ΔcasC*^	*casc* deleted in HGMS9^*ΔchsE1/ΔchsE5/ΔchsE3*^	3-OPC, 3-OPCM, BA	This study
HGMS9^*ΔchsH2/ΔchsH3/chsH2*^	Complementary strain of *chsH2* in HGMS9^*ΔchsH2/ΔchsH3*^	4-AD, BA	This study
HGMS9^*Δltp2/ΔchsH3/ltp2*^	Complementary strain of *ltp2* in HGMS9^*Δltp2/ΔchsH3*^	4-AD, BA	This study
HGMS9^*ΔchsH2/ΔchsH3/chsH3*^	Complementary strain of *chsH3* in HGMS9^*ΔchsH2/ΔchsH3*^	3-OPC, 3-OPCM, BA	This study
HGMS9^*ΔchsE1/ΔchsE5Δ/chsE1*^	Complementary strain of *chsE1* in HGMS9^*ΔchsE1/ΔchsE5*^	4-AD, BA	This study
HGMS9^*ΔchsE1/ΔchsE5/chsE5*^	Complementary strain of *chsE5* in HGMS9^*ΔchsE1/ΔchsE5*^	4-AD, BA, 3-OPC	This study
HGMS9^*ΔchsE1/ΔchsE5/ΔchsE3/chsE3*^	Complementary strain of *chsE3* in HGMS9^*ΔchsE1/ΔchsE5/ΔchsE3*^	3-OPC, 3-OPCM, BA	This study
HGMS9^*ΔechA19/echA19*^	Complementary strain of *echA19* in HGMS9^*ΔechA19*^	4-AD, BA	This study
HGMS9^*ΔfadA5/Δhsd4A/chsH2/ΔfadE30*^	*fadE30* deleted in HGMS9^*ΔfadA5/Δhsd4A/chsH2*^	3-OCO, BA, 3-OCDOM	This study

*Mycolicibacterium* strains were initially cultured in 5 mL of LB medium at 30 °C for a period of two days. This culture was used as the seed culture and subsequently inoculated into 100 mL of fermentation medium. The fermentation medium consisted of the following components: yeast extract (7.5 g/L), glucose (0.5 g/L), NaNO_3_ (2.7 g/L), (NH_4_)_2_HPO_4_ (0.3 g/L), β-cyclodextrin (1.5 g/L), Tween-80 (0.05%, w/v), and phytosterols (5 g/L). The cultures were then incubated at 30 °C with shaking at a speed of 200 rpm.

### Identification of homologue genes of putative mycobacterial phytosterol degradation pathway

Putative phytosterol degradation routes in *Mycolicibacterium* B-3805 were constructed using KEGG mapper [32]. The proteome of HGMS2 (CP011022) [23] was employed to query the annotation data lists for all HGMS2 metabolisms via BlastKoala, utilizing the KEGG Database on *Mycobacterium* (#1763) and *R. jostii* RHA1 (T00382) as references. Among the annotation lists, the phytosterol metabolism pathway (00984) was chosen to construct the phytosterol metabolic pathway, with emphasis placed on the putative enzymes.

### DNA purification and gene knockout experiment

Plasmids utilized in this study are outlined in Table 2. Plasmids were purified using a Plasmid Purification Kit from Tiangen (Beijing, China). The genomic DNAs from B3805 and its mutants were extracted using the Bacterial Genomic DNA Extraction Kit from Tiangen (Beijing, China). The PCR cleanup kit was obtained from Omega BioTek (Hubei, China).

**Table 2:** List of plasmids used in this work

Name	Description	Source
p2NIL-sacB	Vector for gene inactivation in Mycobacterium	Li et al. [15]
p2NIL-sacB-chsH2	p2NIL-sacB derivative for *chsH2* deletion	This study
p2NIL-sacB-ltp2	p2NIL-sacB derivative for *ltp2* deletion	This study
p2NIL-sacB-chsE1	p2NIL-sacB derivative for *chsE1*deletion	This study
p2NIL-sacB-chsE5	p2NIL-sacB derivative for *chsE5* deletion	This study
p2NIL-sacB-fadA5	p2NIL-sacB derivative for *fadA5* deletion	Song et al. [16]
p2NIL-sacB-hsd4A1	p2NIL-sacB derivative for hsd4A1 deletion	Song et al. [16]
p2NIL-sacB-hsd4A2	p2NIL-sacB derivative for hsd4A2 deletion	Song et al. [16]
p2NIL-sacB-chsH3	p2NIL-sacB derivative for chsH3 deletion	This study
p2NIL-sacB-echA19	p2NIL-sacB derivative for echA19 deletion	This study
p2NIL-sacB-chsE3	p2NIL-sacB derivative for chsE3 deletion	This study
p2NIL-sacB-casC	p2NIL-sacB derivative for casC deletion	This study
pMV261	M. neoaurum expression vector	Song et al. [16]
pMV261-chsH2	Recombinant pMV261 for chsH2 expression	This study
pMV261-ltp2	Recombinant pMV261 for ltp2 expression	This study
pMV261-chsE1	Recombinant pMV261 for chsE1 expression	This study
pMV261-chsE5	Recombinant pMV261 for chsE5 expression	This study
pMV261- chsE3	Recombinant pMV261 for chsE3 expression	This study
pMV261- echA19	Recombinant pMV261 for echA19 expression	This study
pMV261- chsH3	Recombinant pMV261 for chsH3 expression	This study

The gene knockout from the *M. neoaurum* HGMS9 genome was performed using a homologous DNA recombination protocol previously described [14–16]. Briefly, the upstream homologous arm consisted of approximately 1 kbp of DNA sequences, including a 60 bp DNA sequence at the beginning of the target gene and the DNA sequence preceding the target gene. The downstream homologous arm comprised approximately 1 kbp of DNA sequences, including a 60 bp DNA sequence in the end of the target gene and the DNA sequence succeeding the end of the target gene. Two DNA homologous arms were amplified and fused with two-step PCR reaction. The resulting PCR products were treated with appropriate restriction enzymes and ligated into pretreated p2NIL-sacB vector. Constructed vector was transformed into *Mycolicibacterium* competent cells using a Gene Electroporation System from SCIENTZ (Linbo, China). Deletion of a target gene was verified through colony PCR screening using two primers that bind to both ends of the coding region of the target gene (Table S1).

For complementation of a deleted gene, the target gene was subcloned into the plasmid pMV261 between the *EcoR* I and *Hind* III sites and subsequently transformed into *Mycobacterium* mutant with the corresponding target gene (Table 3).

**Table 3:** List of the intermediates in phytosterol pathway

Compounds	Steroid name	Abbreviation
1	3-oxo-cholest-4-ene-26-carboxyl-CoA	3-OCS-CoA
2	3-oxo-cholest-4,24-diene-26-carboxyl-CoA	3-OCDS-CoA
3	24-hydroxy-3-oxo-cholest-4-ene-26-carboxyl-CoA	24-HOCO-CoA
4	3-oxo-cholest-4-ene-24-carboxyl-CoA	3-OCO-CoA
5	3-oxo-cholest-4,22-diene-24-carboxyl-CoA	3-OCDO-CoA
6	22-hydroxy-3-oxo-cholest-4-ene-24-carboxyl-CoA	22-HOCO-CoA
7	3,22-dioxo-cholest-4-ene-24-carboxyl-CoA	22-oxo-BNC-CoA
8	3-oxo-4-pregnadiene-20-carboxyl-CoA	3-OPC-CoA
9	3-oxo-4,17-pregnadiene-20-carboxyl-CoA	3-OPDC-CoA
10	17-hydroxy-3-oxo-4-pregnadiene-20-carboxyl-CoA	17-HOPC-CoA
11	4-androstene-3,17-dione	4AD
12	21-hydroxy-20-methylpregn-4-en-3-one	BA
13	3-oxo-4,17-pregnadiene-20-carboxylic acid	3-OPDC
14	3-oxo-4,17-pregnadiene-20-carboxylic methyl ester	3-OPDCM
15	3-oxo-4-pregnadiene-20-carboxylic acid	3-OPC
16	3-oxo-4-pregnadiene-20-carboxylic methyl ester	3-OPCM
17	3-oxo-cholest-4,22-diene-24-carboxylic acid	3-OCDO
18	3-oxochol-4-en-24-carboxylic acid	3-OCO
19	24-norchol-4-ene-3,22-dione	3-OPD
20	3-oxo-cholest-4,22-diene-24-carboxylic methyl ester	3-OCDOM

### Phytosterol biotransformation and analysis of fermentation broth

The phytosterol transformation process by *Mycolicibacterium* strains was conducted for a duration of 7 days, with 1 mL of fermentation broth collected every 24 h throughout the fermentation period. The products present in the fermentation broth were extracted using ethyl acetate at a ratio of 1:1 by thorough mixing. Subsequently, the mixture was centrifuged at 8000 × g for 5 min, and the resulting supernatant was collected. The supernatant was then dried through heating. The dried sample was dissolved in a 40% methanol solution for subsequent HPLC, LC/MS, and NMR assays.

The HPLC and LC/MS analyses were carried out as previously described methods [16]. The one-dimensional ^1^H, one-dimensional ^13^C and two-dimensional ^1^H-^13^ C HSQC NMR spectra were recorded at 298 K on a 500 MHz Bruker AVANCE III. Typically, an NMR sample contained 5–10 mM of compound in deuterated chloroform (CDCl_3_) solvent.

## Results

### The putative main route for the conversion of phytosterols to 4-AD in *M. neoaurum* HGMS strain

This study investigates the relationship between the main phytosterol side-chain degradation route and its associated secondary routes during the 4-AD accumulation in the HGMS strain. Our genomic analysis (see *Materials and methods*) indicates that phytosterol side-chain degradation in HGMS strain involves three cycles of β-oxidation, following the initial oxidization by cholesterol oxidases (ChoMs) and 3β-keto-steroid reductase (HSD) (Scheme I, Step 1). However, key enzymes catalyzing Steps 2–13 in Scheme I remain largely uncharacterized.

Therefore, we systematically annotated the roles of these putative enzymes in the main phytosterol side-chain degradation route. To confirm the specificity of each putative enzyme for β-oxidation of phytosterols, we investigated them on reverse order, beginning from the final step (Step 13) and proceeding backward to Step 2 where feasible.

### The reactions in steps 12 and 13 were catalyzed by the hydratase ChsH1/H2 complexes and aldolase Ltp2/ChsH2complexes

**Fig. 1:**
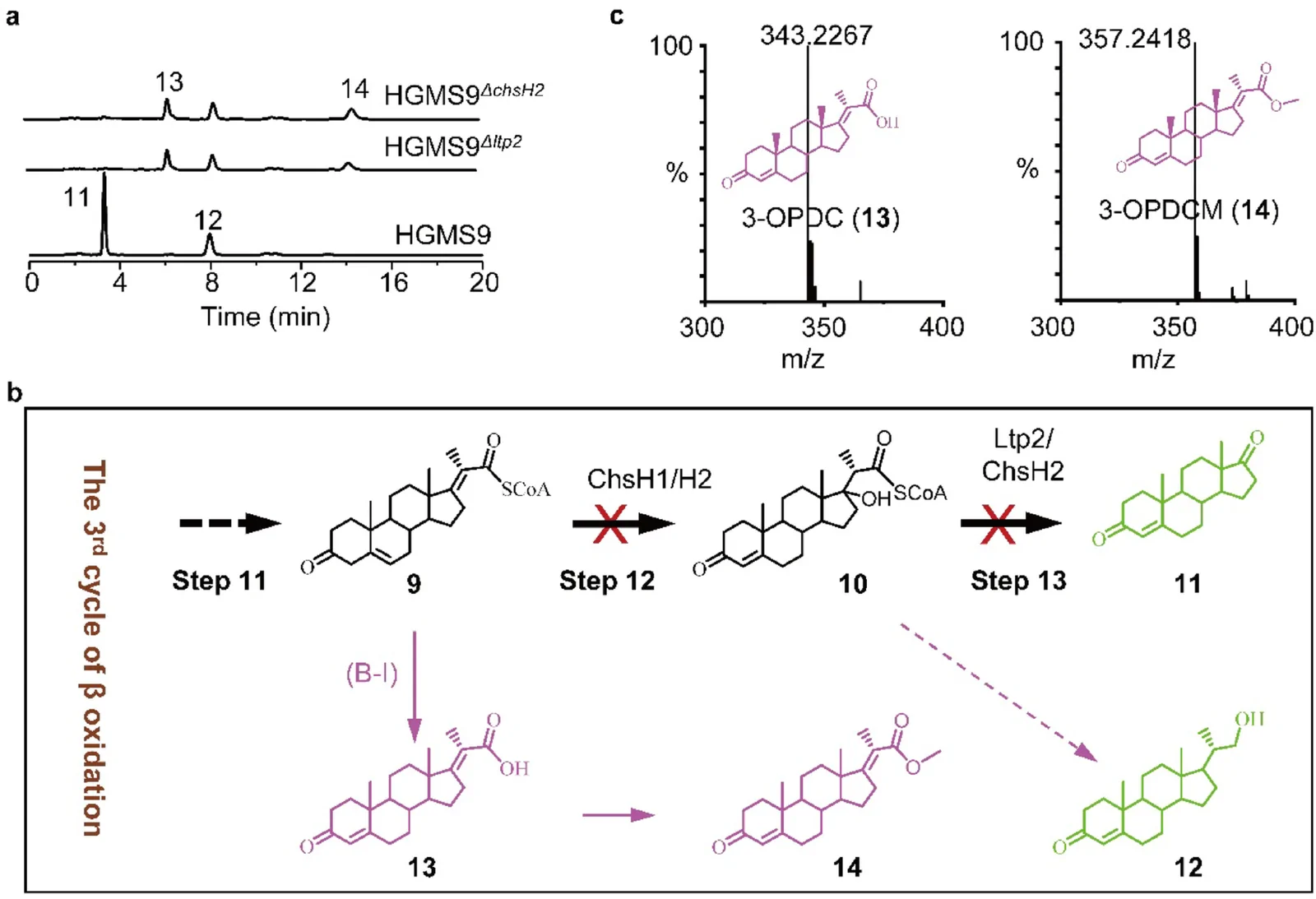
Knocking out the *ltp2* or *chsH2* genes in HGMS9 promotes a secondary route. **a.** Comparison of HPLC profiles of HGMS9^*Δltp2*^ and HGMS9^*ΔchsH2*^ with HGMS9. **11**: 4-AD; **12**: BA; **13**: 3-OPDC and **14**: 3-OPDCM. **b**. Mass spectra of Compounds **13** and **14**. **c**. B-I secondary route (*pink arrows*) is derived from the main route (*black arrows*) to accumulate 4-AD after the dysfunction of the *ltp2* or *chsH2* genes

To streamline our investigation, we first eliminated the accumulation of ADD and 9OH-AD by knocking out the *kstD211*,* kshA395* and *kshB122* in B-3805 using our previous protocol [15]. This generated a mutant strain, designated HGMS9 (Table 1). HPLC analysis of HGMS9 fermentation extracts revealed that this strain accumulated two significant intermediates, 4-AD (Compound **11)** and BA (Compound **12**), during phytosterol degradation (Fig. 1a, the bottom curve).

**Scheme 1:**
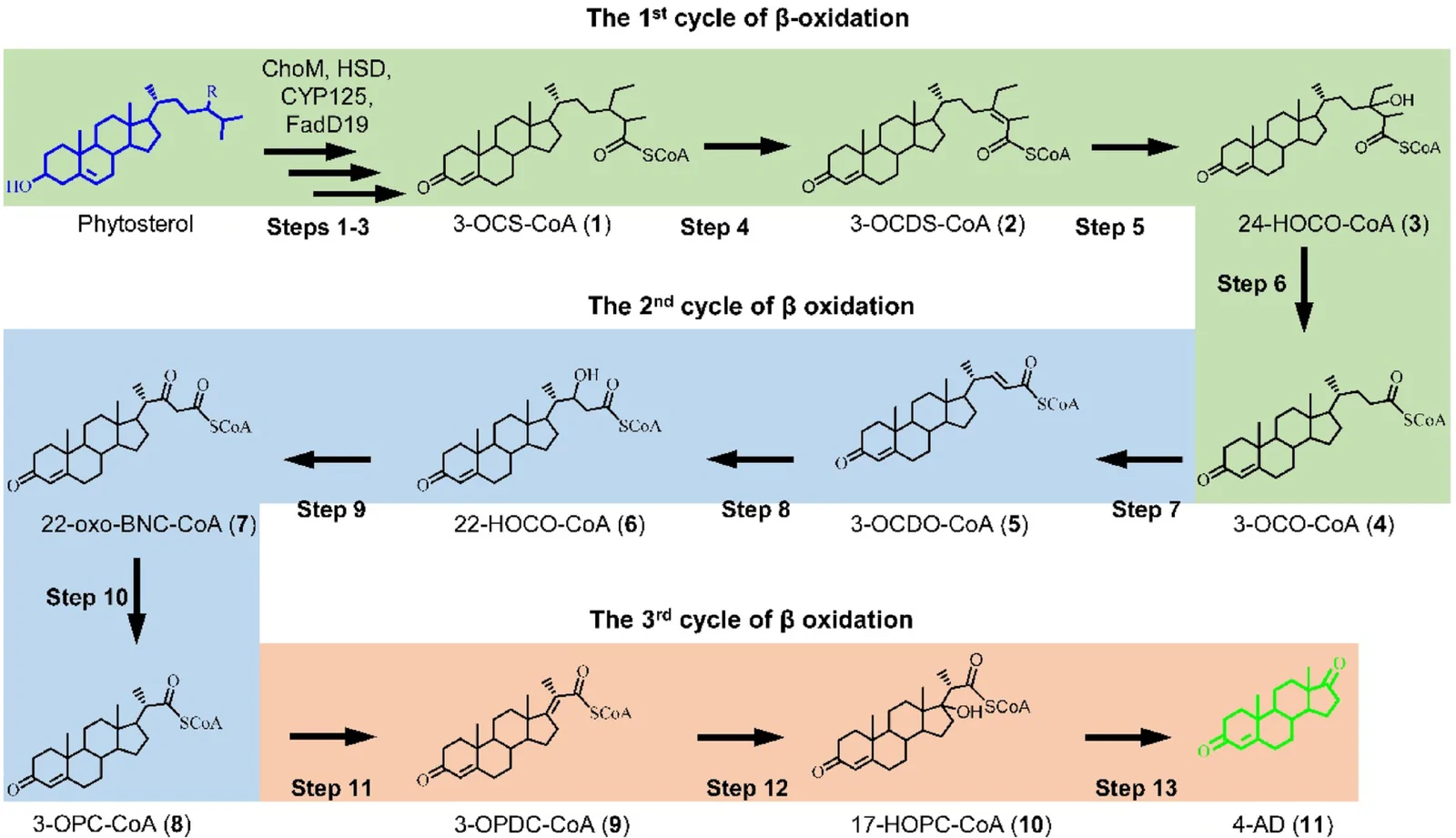
Mycolicibacterial phytosterol side-chain degradation to accumulate 4-AD through 12 steps of three cycles of β-oxidation. This scheme was summarized based on the references [4, 6, 14, 17–26]. *Blue*: substrates, i.e., phytosterols; *black*: intermediates (Compounds **1**–**10**). *Green*: 4-AD (Compound **11**); green box: the 1st cycle of β-oxidation; cyan box: the 2nd cycle of β-oxidation; brown box: the 3rd cycle of β-oxidation. The two leaving groups are CH_3_CH_2_CO-SCoA and CH_3_CO-SCoA during three cycles of β-oxidation. Thick black arrows represent main route of phytosterol side-chain degradation. Question marks refer to unknown or unsured enzymes

Based on Scheme 1 and Fig. 1b, the enzymes ChsH2 and Ltp2 are predicted to catalyze the final two steps (Steps 12–13) of 4-AD production in HGMS9. To define their role in the third cycle of β-oxidation, we knocked out the *chsH2* and *ltp2* genes from HGMS9. HPLC analysis of the resulting single-gene knockout mutants (HGMS9^*Δltp2*^ and HGMS9^*ΔchsH2*^) showed nearly identical profiles, revealing the accumulation of three major intermediates (Fig. 1a, two top curves).Compared to the parent HGMS9 strain, 4-AD (Compound **12**) absent, while BA (Compound **12**) remained. Two new peaks extracts were identified by mass spectrometry as 3-OPDC (Compounds **13**) and 3-OPDCM (Compound **14**) (Fig. 1c). The NMR spectrometric profile for Compounds **13** is shown in Fig. S1. The similar HPLC profiles of the two mutants suggest a close structural and functional association between the Ltp2 enzyme and the ChsH1/H2 enzyme complexes. The disappearance of 4-AD (Compound **11**) and the concurrent appearance of 3-OPDC (Compound **13**) and 3-OPDCM (Compound **14**) demonstrate that both the ChsH1/H2 complexes and Lpt2 are essential for the final β-oxidation cycle, specifically for Steps 12 and 13. We therefore designated a secondary route as the B-I branch route, which commences from Compound **9** (Fig. 1b).

### The reaction in step 11 was catalyzed by either the ChsE1/E2 or ChsE4/E5 dehydrogenase complexes initiating the third cycle of β-oxidation

**Fig. 2:**
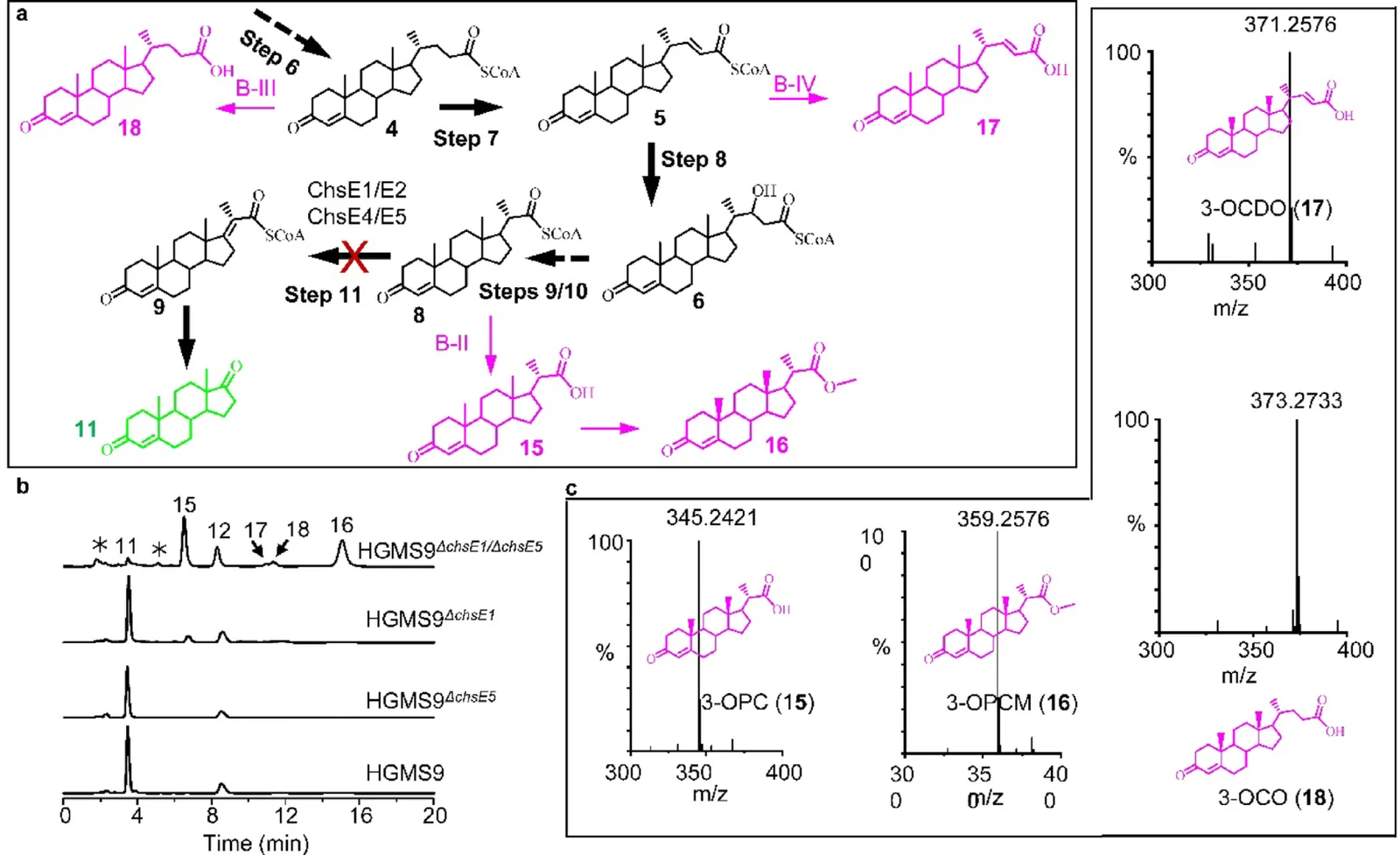
Knocking out the *chsE1* and *chsE5* genes promotes three secondary routes. **a**. Comparison of HPLC profiles of the fermentation broth extracts from the double mutant with disabling both *chsE1* and *chsE5* genes with the single mutation and HGMS9. **15**: 3-OPC; **16**: 3-OPCM. **17**: 3-OCDO; **18**: 3-OCO; Asterisk: unknown impurities from fermentation media. **b**. Mass spectra of Compounds **15 16**, **17** and **18**. **c**. Three secondary routes, B-II, B-III and B-IV, start from the intermediates **8**, **4** and **5**, respectively, of the main route during the accumulation of 4-AD (Compound **11**)

Guided by Scheme I and Fig. 2a, we examined the role of the ChsE1/E2 and ChsE4/E5 enzyme complexes in phytosterol side-chain degradation using mutants lacking either *chsE1* or *chsE5* genes (HGMS9^*ΔchsE1*^ and HGMS9^*ΔchsE5*^). HPLC analysis revealed minimal differences in the metabolite profiles of these single mutants compared to the parent HGMS9 strain, with one exception that the HGMS9^*ΔchsE1*^ mutant accumulated an additional intermediate (Compound **15**, Fig. 2b). This suggests that ChsE1 and ChsE5 are isoenzymes with similar activities, capable of compensating for each other’s loss.

In contrast, the double mutant HGMS9^*ΔchsE1/ΔchsE5*^ exhibited a dramatic phenotypic shift. The accumulation of 4-AD (Compound **11**) was significantly reduced while levels of BA (Compound **12**), and two other Compounds (**15** and **16**, Fig. 2b) appeared. They were identified via mass spectrometric and NMR spectrometry, as 3-OPC and 3-OPCM, respectively (Fig. 2c, Fig. S2). These data demonstrate unequivocally that the ChsE1/E2 and ChsE4/E5 complexes catalyze the reaction immediately following Compound **8** in the final β-oxidation cycles. Consequently, we identified Compounds **15** and **16** as products of a secondary route originate from Compound **8**, which we designated the B-II secondary route (Fig. 2a).

Furthermore, we detected two additional trace intermediates, designated Compound **17** and Compound **18** (Fig. 2a and Supplementary materials). They were identified by Mass and NMR spectrometry as 3-OCDO and 3-OCO, respectively. Based on their structures, we propose that Compound **17** derives from Compound **5**, and Compound **18** from Compound **4** (Fig. 2a). We have therefore defined the formation of Compound **18** and Compound **17** as the B-III and B-IV secondary routes, respectively. The accumulation of these early intermediates suggests that the ChsE1/E2 and ChsE4/E5 enzyme complexes may be also involved in initiating the second β-oxidation cycle (Step 7), exerting a distal effect on the B-III and B-IV branch routes. This implies the existence of additional, as-yet-unidentified isoenzymes that can partially compensate for the loss of ChsE1/E2 and ChsE4/E5 in the second cycle.

### The reaction in steps 9 and 10 catalyzed by the acyl-CoA thiolase FadA5 and acyl-CoA dehydrogenase Hsd4A are two limited steps in the 4-AD accumulation

**Fig. 3:**
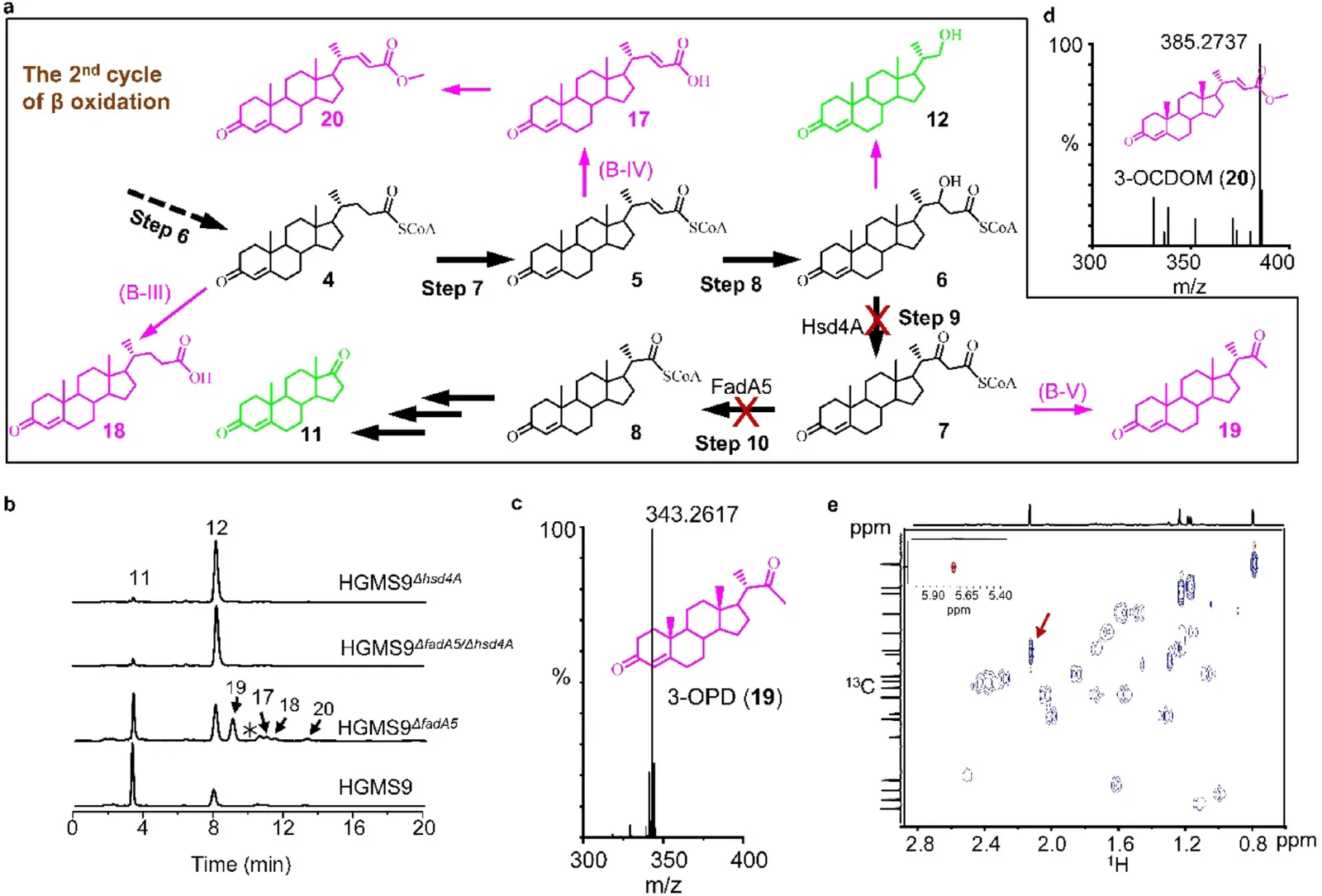
Knocking out the *fadA5* gene promotes the fifth secondary route. **a.** Comparison of HPLC profiles of the fermentation broth extract from the single mutant disabling the *fadA5* gene with HGMS9^*Δhsd4A*^, HGMS9^*Δhsd4A/ΔfadA5*^ and HGMS9. **19**: 3-OPD and **20**: 3-OCDOM **b**. Mass spectrum of Compound **19**. **c**. The ^1^H-^13^ C HSQC spectrum of Compound **19**. **d**. Mass spectrum of Compound **20**. **e**. The fifth secondary route (B-V) appears after deleting the *fadA5* gene, while the B-III and B-IV secondary routes still exist

As outlined in **Scheme I** and Fig. 3a, the reactions in Steps 9 and 10 are catalyzed by the dehydrogenases Hsd4A and the thiolase FadA5, respectively. Deletion of the *fadA5* gene in the HGMS9 strain resulted in increased accumulation of BA (Compound **12**) and the appearance of two new metabolites, designated Compounds **19** and **20** (Fig. 3b). Mass spectrometric and NMR analyses identified these compounds as 3-OPD and 3-OCDOM (Fig. 3c–e and Supplementary Materials).

To investigate the functional interplay between the enzyme FadA5 and Hsd4A, we knocked out the *hsd4A* gene in the HGMS9^*ΔfadA5*^ mutant. This double knockout (HGMS9^ΔfadA5/Δhsd4A^) redirected the metabolism towards the accumulation of BA (Compound **12**), with only a trace amount of 4-AD (Compound **11**) remaining (Fig. 3b). Notably, Compounds **17**, **18**, **19** and **20** became undetectable. The HPLC profile of the fermentation extract from this double mutant was nearly identical to that of the single mutant HGMS9^*Δhsd4A*^, confirming that the reaction in Step 9 acts as a critical switch controlling metabolic flux [16]. These results indicate that the accumulation of 3-OPD (Compound **19**) originates from Compound **7**, defining a novel secondary route. We have designated this secondary as the B-V branch route (Fig. 3a).

Furthermore, the metabolic redirection observed in the HGMS9^*Δhsd4A*^ single mutant confirms that BA formation constitutes a secondary route originating from Compound **6** (Fig. 3a). The enzymes in this secondary route are highly active, effectively outcompeting other routes and preventing the accumulation of Compounds **17**, **18**, **19**, and **20**. The persistence of a small amount of 4-AD in the HGMS9^*Δhsd4A*^ suggests the potential existence of less active FadA5 and Hsd4A isoenzymes in HGMS9 (Fig. 3b).

### The reaction in step 8 is catalyzed by the same enzyme complexes as those of Steps 12 and 13 except for ChsH3

**Fig. 4:**
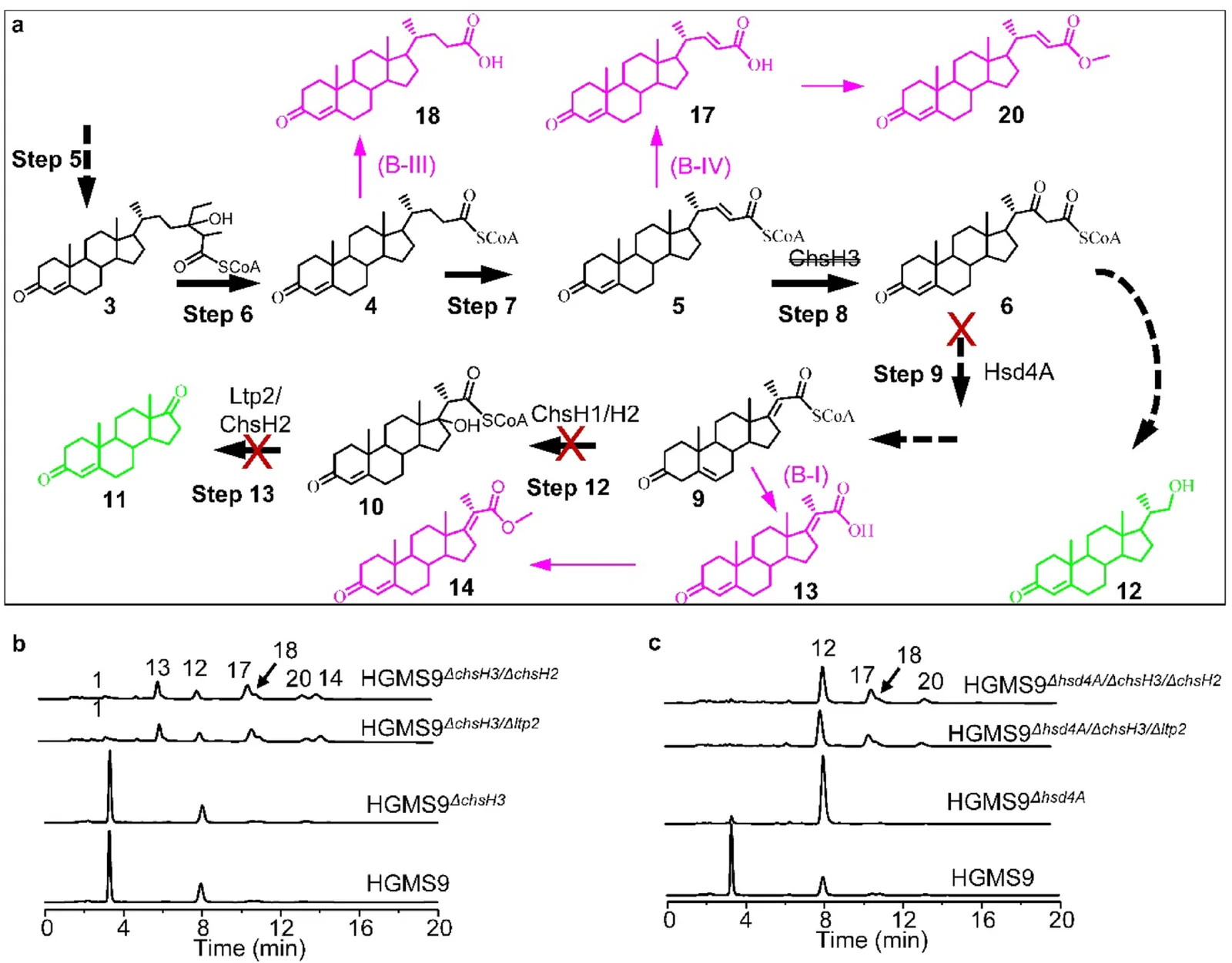
The *chsH3* gene is nonessential in HGMS9. **a**. Comparison of HPLC profiles of the fermentation broth extracts from the mutants disabling the *chsH3* gene in combination the *lpt2*- or *chsH2-*genes with that of HGMS9. **b**. Comparison of HPLC profiles of the fermentation broth extracts from the mutants disabling the *chsH3* gene in combination with the *ltp2*-, *chsH2*- and *hsd4A*- genes with that of HGMS9. **c**. Multiple genes including *chsH3*, *hsd4A*, *ltp2* and *chsH2* are relevant to the initiation of the B-1, B-III and B-IV secondary routes

Initial analysis based on **Scheme I** and Fig. 4a, indicated that the reaction in Step 8 could be catalyzed by ChsH3. To test its role in phytosterol side-chain degradation, we analyzed the HPLC profiles of fermentation extracts for the HGMS9^*ΔchsH3*^ mutant. Its HPLC profile was largely unchanged from that of HGMS9 (Fig. 4b). In contrast, double mutants lacking chsH3 in combination with chsH2 or ltp2 exhibited profoundly different HPLC profiles (Fig. 4b, top two curves). These profiles were distinct from those of the single mutants and featured the accumulation of 3-OPDC (**13**), 3-OPDCM (**14**), BA (**12**), 3-OCDO (**17**), 3-OCO (**18**), and 3-OCDOM (**20**).

We further generated two triple mutants the HGMS9^*Δhsd4A*^ strain by additionally deleting both the *chsH3*/*chsH2* and *chsH3/ltp2* gene pairs. The metabolites profiles of the resulting HGMS9^*Δhsd4A/ΔchsH3/ΔchsH2*^ and HGMS9^*Δhsd4A/ΔchsH3/Δltp2*^ mutants were identical. Both accumulate BA (**12**), 3-OCDO (**17**), 3-OCO (**18**) and 3-OCDOM (**20**) while 3-OPDC (**13**) and 3-OPDCM (**14**) were absent (Fig. 4c). These findings demonstrate that the appearance of Compounds **17**, **18** and **20** is a direct consequence of inactivating the ChsH1/H2/Ltp2 enzyme complexes.

The deletion of the *chsH3*/*ltp2* and *chsH3*/*chsH2* gene pairs significantly impeded cell growth compared to HGMS9 and HGMS9^*Δhsd4A*^ (Fig. S3a). This growth defect consequently led to a notable reduction in BA (**12**) yield, which dropped to 0.75 g/L and 0.84 g/L in the respective triple mutants from 2.01 g/L in HGMS9^*Δhsd4A*^ (Fig. S3b). This confirms that the ChsH1/H2/Ltp2 enzyme complexes are essential for the second round of the β-oxidation cycles.

Therefore, the ChsH3 enzyme acts upstream of Hsd4A, as evidenced by the substantial accumulation of BA (**12**) in HGMS9^*Δhsd4A*^. in the phytosterol degradation route in HGMS9, given the substantial accumulation of BA (**12**) in HGMS9^*Δhsd4A*^. The critical reaction in Step 8, like those in Steps 12 and 13, is primarily catalyzed by the ChsH1/H2 and Ltp2/ChsH2 complexes, not ChsH3.

### The acyl-CoA hydratase ChsE3 and acyl-CoA dehydrogenase CasC are not essential for phytosterol side-chain degradation but for HGMS9 cell growth

**Fig. 5:**
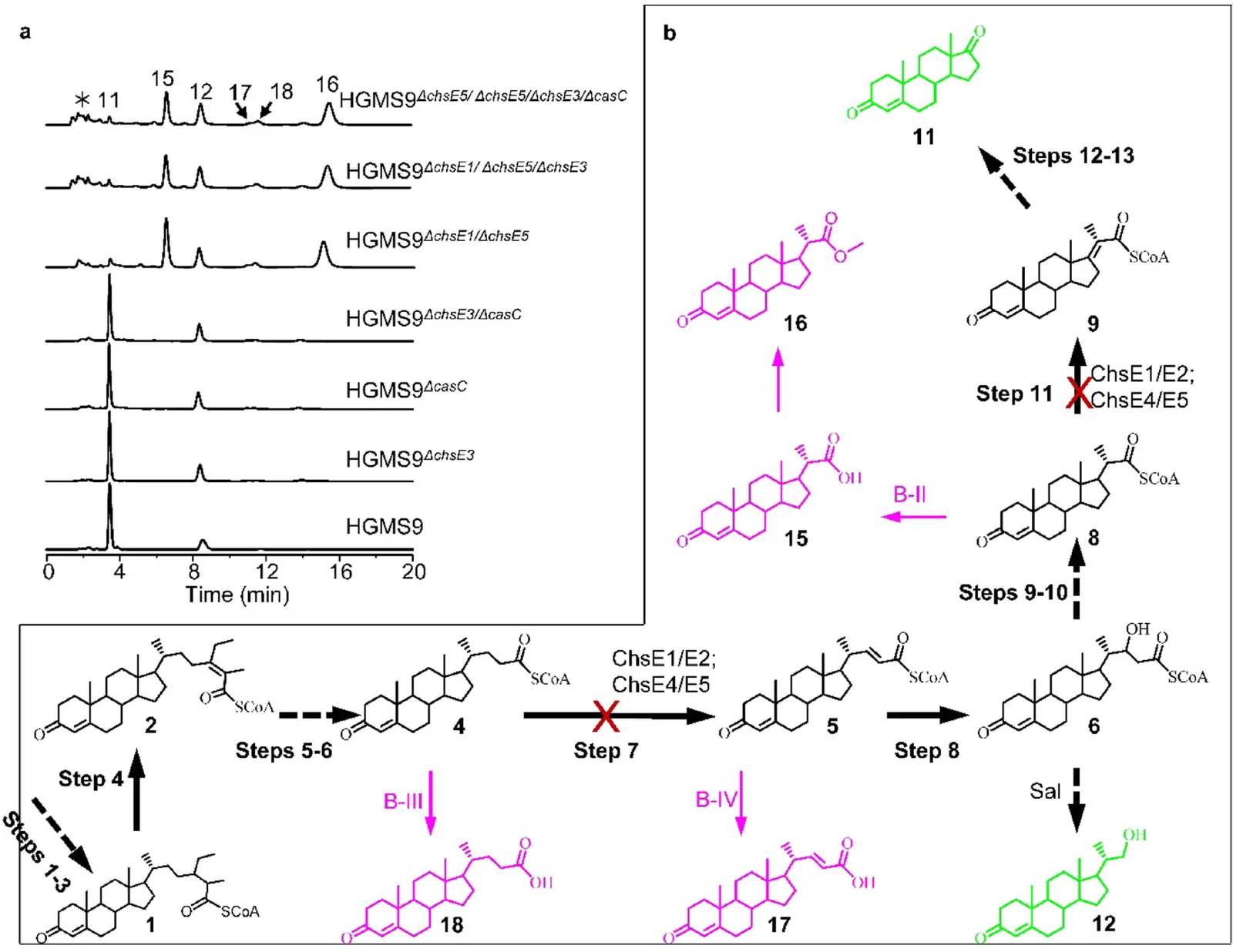
The *chsE3* and *casC* genes are nonessential in HGMS9. **a**. Comparison of HPLC profiles of the two single mutants with disabling the *chsE3* and *casC* genes with those of HGMS9^*ΔchsE3*^, HGMS9^*ΔcasC*^, HGMS9^*ΔchsE3/ΔcasC*^, HGMS9^*ΔchsE1/ΔchsE5*^, HGMS9^*ΔchsE1/ΔchsE5/ΔchsE5*^, HGMS9^*ΔchsE1/ΔchsE5/ΔchsE3/ΔcasC*^ and HGMS9. **b**. The B-II, B-III and B-IV secondary routes remain in the fermentation media of the double mutant HGMS9^*ΔchsE1/ΔchsE5*^

To investigate the potential involvement of ChsE3 and CasC in Step 7, as proposed in **Scheme I**, we constructed *chsE3* and *casC* knockout mutants from HGMS9. The HPLC profiles of the fermentation medium extracts from the single HGMS9^*Δ*chsE3^, HGMS9^*Δ*casC^ and double (HGMS9^*Δ*chsE3*/Δ*casC^) mutants showed no significant differences from that of the HGMS9 strain (Fig. 5a). The result suggests that either ChsE3 or CasC enzymes is associated with the phytosterol side-chain degradation in HGMS2.

The role of ChsE3 and CasC was further investigated by generating higher order mutants by deleting the *chsE3* and *casC* genes from HGMS9ΔchsE1/ΔchsE5. The resulting triple mutant namely HGMS9^*ΔchsE1/ΔchsE5/ΔchsE3*^ and tetramutant (HGMS9^*ΔchsE1/ΔchsE5/ΔchsE3/ΔcasC*^) strains exhibited HPLC profiles nearly identical to the original double mutant (Figs. 2a and 5a). Furthermore, the tetramutant accumulated the same intermediates including 3-OPC (**15**), 3-OPCM (**16**), 3-OCDO (**17**), 3-OCO (**18**), and BA (**12**) as the HGMS9^*ΔchsE1/ΔchsE5*^ strain. These results confirm that the ChsE1/E2 and ChsE4/E5 enzyme complexes are also involved in the reaction at Step 7, which initiates the second cycle of β-oxidation (Fig. 5b). However, the data indicates that both complexes have a significantly higher catalytic activity for Step 11 than for Step 7.

Bioinformatic analysis suggested that the dehydrogenase FadE30 known to degrade secophenol, is a candidate for catalyzing Step 7 in the phytosterol side-chain cleavage route. To test this, we deleted the *fadE30* gene from the HGMS9^*ΔHsdA1/ΔHsdA4/ΔchsH2/ΔFadA5*^ strain. This deletion resulted in a marked increase in the accumulation of Compound 18 increased, indicating a blockage at Step 7. This finding confirms that that FadE30 is an isoenzyme of ChsE4/E5 (Fig. S4).

We further investigated the roles of the ChsE3 and CasC through geneic deletion. The single or simultaneous deletion of *chsE3* and *casC* had no significant effect on cell growth. In contrast, the growth of the HGMS9^*ΔchsE1/ΔchsE5/ΔchsE3*^ mutant was severely impaired (Fig. S4a). Deleting *chsE3* in the HGMS9^*ΔchsE1/ΔchsE5*^ further inhibited growth, and the subsequent deletion of the *casC* gene from this triple mutant did not alter this low growth rate (Fig. S4a). This demonstrates that the ChsE3 and CasC enzymes are not involved in the phytosterol side-chain degradation in the HGMS2 strain.

Consistent with the growth defect, metabolite yields were significantly reduced in these mutants (Fig. S4b-d). The HGMS9^*ΔchsE1/ΔchsE5/ΔchsE3/ΔcasC*^ strain produced only 0.08 g/L of BA (Compound **12**), 0.18 g/L of OPC (Compound **15**), and 0.20 g/L of 3-OPCM (Compound **16**) from phytosterols. The HGMS9^*ΔchsE1/ΔchsE5*^ strain also only accumulated 0.13 g/L of BA (Compound **12**), 0.32 g/L of 3-OPC (Compound **15**), and 0.22 g/L of 3-OPCM (Compound **16**) during phytosterol transformation.

### EchA19 catalyzes a rate-limiting step in the initial round of β-oxidation

**Fig. 6:**
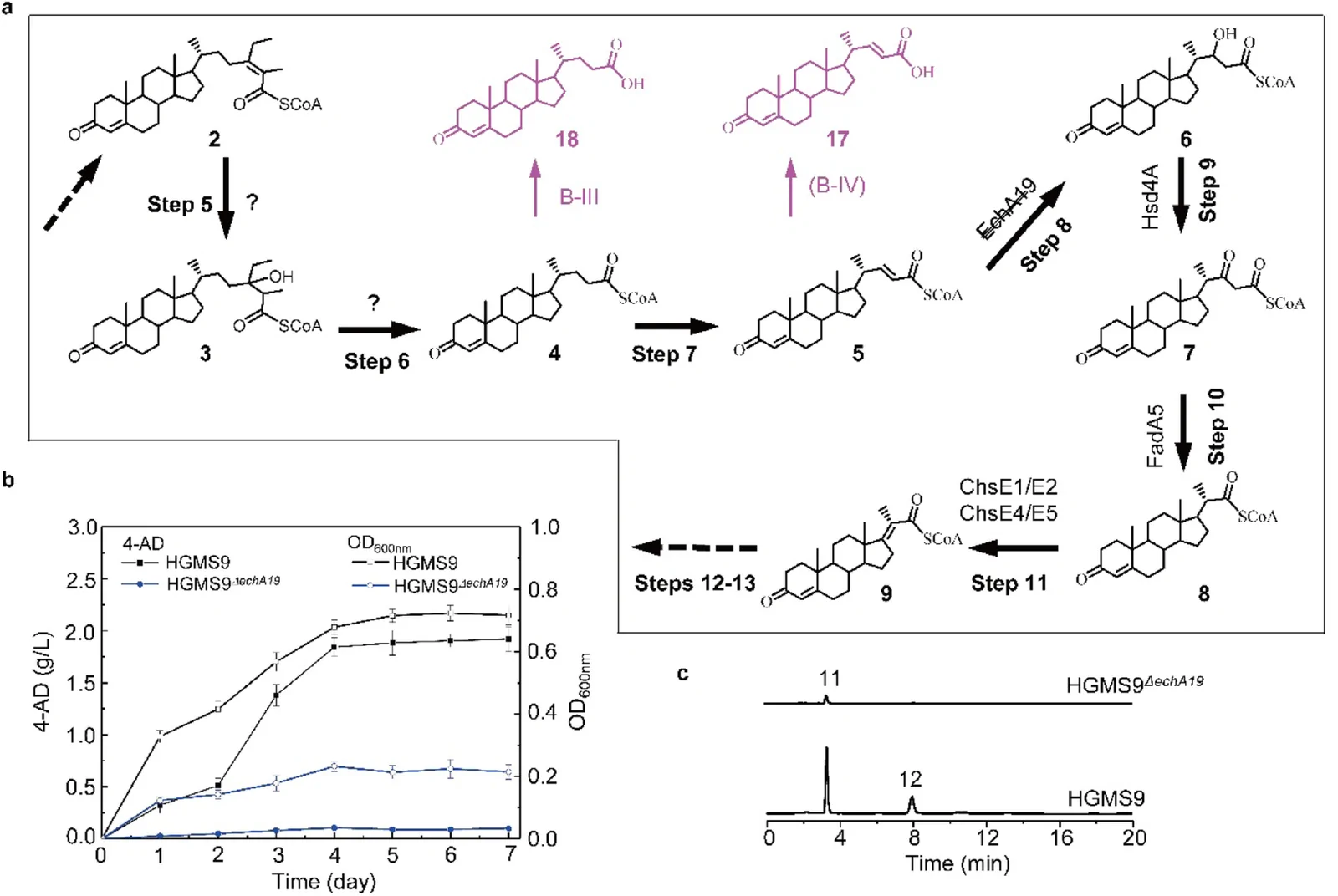
Knocking out the *echA19* gene exhibits no effect on the 4-AD accumulation in HGMS2, but on cell growth. **a.** Comparison of the 4-AD concentration and growth of the single mutant disabling the *echA19* gene with HGMS9. **b.** Comparison of HPLC profiles of the fermentation broth extracts from the single mutant with disabling the *echA19* gene with HGMS9. **c**. The B-III and B-IV secondary routes remain in the fermentation media of the mutant HGMS9^*ΔechA19*^

We hypothesized that deleting echA19 gene, which encodes an acyl-CoA hydratase, would block the conversion of 3-OCDO-CoA (5) to 22-HOCO-CoA (6) and divert metabolic flux towards the B-III and B-IV secondary routes (**Scheme I**, Fig. 6a). Consistent with this, deletion of echA19 in HGMS9 significantly impaired cell growth and phytosterol degradation (Fig. 6b). The yield of the main product, 4-AD (Compound 11), plummeted by 94.7% (20-fold) to 0.1 g/L, though it remained the dominant product detected (Fig. 6c).Contrary to our initial hypothesis, we did not detect the accumulation of 3-OCDO (Compound **17**) and 3-OCDOM (Compound **20**), which would be expected if the final β-oxidation steps were blocked. This suggests that the EchA19 enzyme primarily catalyzes the hydration of 3-OCDS-CoA (Compound **2**) to 24-HOCO-CoA (Compound **3**) in the first round of the β-oxidation cycles. rather than the reaction from Compounds **5** to **6** in the last two rounds of the β-oxidation cycles (**Scheme I**). Given the severe impact of its deletion on the entire route, we conclude that the reaction from Compound **2** to Compound **3**, catalyzed by EchA19, is a rate-limiting step for phytosterol degradation.

### Complementation experiments rule out polar effects in the HMGS2 phytosterol degradation pathway

To confirm the specific functions of the deleted genes in HGMS9 and rule out potential polar effects, we performed complementation experiments by reintroducing the *chsE1*,* chsE3*,* chsE5*,* chsH2*,* chsH3*,* ltp2*, and *echA19* genes into their respective mutant strains using the pMV261 plasmid (Table 2).

The functional restoration of these genes successfully rescued the mutant phenotypes. Complementation of *chsH2* in HGMS9^*ΔchsH3/ΔchsH2*^ or *ltp2* in HGMS9^*ΔchsH3/Δltp2*^ restored 4-AD (**11**) production to the level of the HGMS9^*ΔchsH3*^ single mutant. Thus, the HGMS9^*ΔchsH3/ΔchsH2/chsH3*^ strain ceased accumulating the intermediates 3-OCDO (**17**), 3-OCO (**18**), and 3-OCDOM (**20**) (Fig. S5a). Similarly, complementing *chsE1* or *chsE5* in the HGMS9^*ΔchsE1/ΔchsE5*^ double mutant yielded phenotypes resembling the HGMS9^*ΔchsE5*^ or HGMS9^*ΔchsE1*^ single mutants, respectively, with 4-AD as the main product (Fig. S5b). Furthermore, reintroducing *chsE3* into the HGMS9^*ΔchsE1/ΔchsE5/ΔchsE3*^ triple mutant restored phytosterol conversion and significantly enhanced the production of BA (**12**), 3-OPC (**15**), and 3-OPCM (**16**) (Fig. S5b), while complementation of *echA19* in HGMS9^*ΔechA19*^ fully restored the wild-type phenotype (Fig. S5c). Collectively, the successful reversal of all mutant defects through gene-specific complementation definitively confirms the essential and direct roles of these enzymes in the phytosterol side-chain degradation route.

## Discussion

**Scheme 2:**
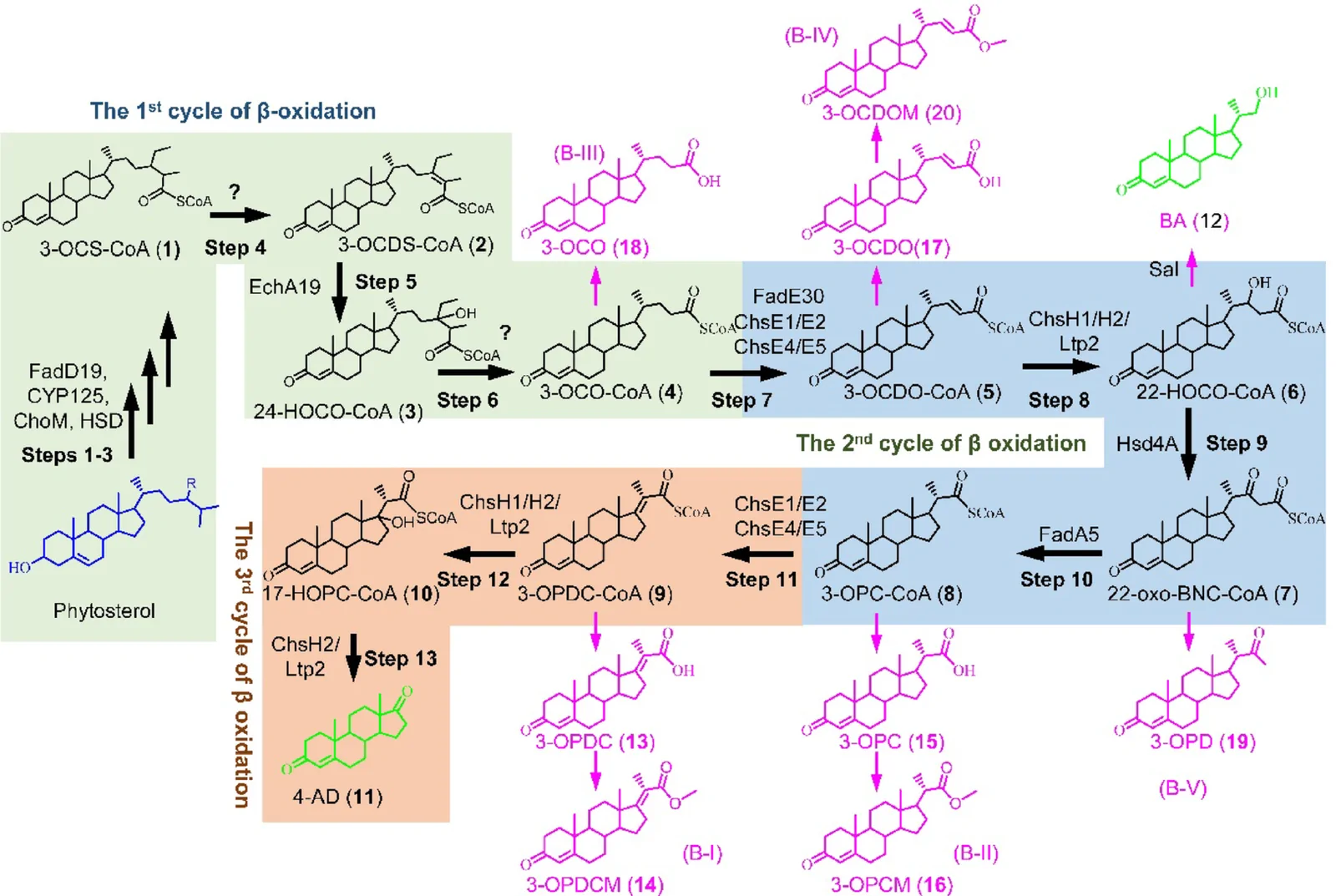
Updated phytosterol side-chain degradation routes in HMGS or 3805. *Blue*: phytosterols; *green*: 4-AD (Compound **11**) and BA (Compound **12**); *black*: intermediates. *pink*: secondary routes. Question marks: unassigned enzymes. Blue box: the 1st cycle of β-oxidation; cyan box: the 2nd cycle of β-oxidation; brown box: the 3rd cycle of β-oxidation. Five secondary routes are depicted in pink. Thick black arrows represent main route of phytosterol side-chain degradation and thin black arrows represent secondary route, branching from the main route

Through a multi-gene knockout approach, we identified key enzymes responsible for 11 specific steps in the main route of the three β-oxidation cycles for phytosterol side-chain degradation in the HGMS or B-3805 (Scheme 2). The enzymes for Steps 1–3 include ChoM and HSD, which oxidizes the 3β-hydroxyl group, while CYP125 and FadD19 oxidize the terminal end of the side-chain [14, 16, 33]. In the first β-oxidation cycle, EchA19 is exclusively involved in Step 5. This contracts with other mycolicibacterial strains where EchA19 is responsible for the second and third β-oxidation cycles [34, 35]. ChsE1/E2 and ChsE4/E5 complexes catalyze similar steps to initiate the second and third cycles (Steps 7 and 11, Scheme 2). The FadE30 enzyme can initiate the second cycle, functioning complementarily to the ChsE4/E5 complexes at Step 7. The ChsH1/H2/Ltp2 complexes catalyze equivalent steps in the second and third cycles (Steps 8 and 12, Scheme 2). Furthermore, Hsd4A and FadA5 are specific for Steps 9 and 10 in the second cycle. The ChsH2/Ltp2 complexes catalyze Step 13 to finalize the side-chain degradation, yielding the product 4-AD (**12**). Notably, while ChsE3 [36] and CasC [37] were not involved in phytosterol side-chain degradation in the HGMS or B-3805 strain (Fig. 5), the enzymes specific for Step 4 and Step 6 remain unidentified through current multi-gene knockout approach.

Gene knockout strategies in the HGMS strain identified five secondary routes (B-I to B-V, Scheme 2) that branch from the main phytosterol side-chain degradation route. Key enzymes that initiate these branches were pinpointed, leading to the accumulation of distinct C22, C23, and C24 intermediates. The final products of these secondary routes are methyl esters (B-I, B-II, B-IV), a fatty acid (B-III), and a keto compound (B-V). As illustrated in Scheme 2, the five secondary routes are connected to the main route at Compounds **4**, **5**, **7**, **8**, and **9**, respectively The enzymes responsible for initiating these secondary routes belong to thioesterases. However, the presence of all thioesterases, including the Sal enzyme that initiate the accumulation of BA (Compound **12**), has not been confirmed yet in HGMS or B-3805, necessitating further investigation [38].

In conclusion, our study elucidates the metabolic pathway for phytosterol side-chain degradation in mycolicibacteria. These findings provide a framework for identifying by-product in the production of 4-AD (Compound **11**) and BA (Compound **12**) and lay the foundation for developing more efficient industrial strains. Using synthetic biology strategy, these strains can be engineered to produce key intermediates for the steroid pharmaceutical industry.

## Supplementary Information

Below is the link to the electronic supplementary material.

Supplementary Material 1.

## Data Availability

No datasets were generated or analysed during the current study.

## Abbreviations

3-OCO: 3-oxochol-4-en-24-carboxylic acid
3-OPDC: 3-oxo-4,17(20)-pregnadiene-20-carboxylic acid
3-OPCM: 3-oxo-4-pregnadiene-20-carboxylic methyl ester
3-OPDCM: 3-oxo-4,17-pregnadiene-20-carboxylic methyl ester
4-AD: 4-androstene-3,17-dione
9OH-AD: 9α-hydroxy-4-androstenedione
22-HOCO-CoA: 22-hydroxy-3-oxo-cholest-4-ene-24-carboxyl-CoA
22-oxo-BNC-CoA: 3,22-dioxo-cholest-4-ene-24-carboxyl-CoA
ADD: 1,4-androstadienedione
BA: 21-hydroxy-20-methylpregn-4-en-3-one
ChsE1/E2: Cholesterol acyl-CoA dehydrogenase E1/E2
ChsE3: Cholesterol acyl-CoA dehydrogenase E3
ChsE4/ChsE5: cholesterol acyl-CoA dehydrogenase E4/E5
ChsH1/ChsH2: cholesterol acyl-CoA hydratase H1/H2
ChoM: Mycolicibacterial cholesterol oxidases
HSD: 3β-keto-steroid reductase
Ksh: 3-ketosteroid-9α-hydroxylases
Kstd: 3-ketosteroid-Δ^1^-dehydrogenase
