# Supplementary material for: Uncovering shared enzymes and secondary routes across β-oxidation cycles in Mycolicibacterial phytosterol side-chain degradation
Source: Microb Cell Fact. 2026 Aug 12;25:234. doi: 10.1186/s12934-026-03087-7 (PMC13753135; doi:10.1186/s12934-026-03087-7)
Supplement: Supplementary file 1 [file 12934_2026_3087_MOESM1_ESM.pdf]

Supporting Information for

Uncovering shared enzymes and branch pathways across β-oxidation cycles in *Mycolicibacterial* phytosterol side-chain degradation

Qianqian Ma[1,2,#], Jianxin He[2,#], Yupan Zhou[3], Xirenayi Tuergong[4], Ye Wang[1,2], Jiaxue Chang[1], Jiayi Wang[1], Alimire Ruzi[1], Xiyao Cheng[3,*], Shikui Song[3,*] and Zhengding Su[1,2,*]

[1]School of Pharmaceutical Sciences and Institute of Materia Medica, Xinjiang University, Urumqi, Xinjiang, 830017, China

[2]Laboratory of Protein Engineering and Biopharmaceutical Sciences, Hubei University of Technology, Wuhan 430068, China

[3]School of Light Industry and Food Engineering, Guangxi University, Nanning, Guangxi, 530004, China

[4]Xinjiang Key Laboratory of Special Species Conservation and Regulatory Biology, College of Life Sciences, Xinjiang Normal University, Urumqi, 830054, Xinjiang, China

# These authors equally contribute to this work.

[*]**To whom correspondence should be addressed:** Zhengding Su, Email: zhengdingsu@hbut.edu.cn, ORCID iD: 0000-0003-3558-001X or Xiyao Cheng, Email: xiyaocheng@gxu.edu.cn, ORCID iD: 0000-0003-2161-1743 or Shikui Song, Email: sksong@xjnu.edu.cn, ORCID iD: 0009-0005-9795-4859.

Table S1. Primers used in this study

Targeted gene	Primers	Sequence	Note
chsE1	chsE1-D-F	GCTCTAGAATAGCCATGTACATCGAACTG	*Xba* I
	chsE1-D-R	CCCAAGCTTCTGCCAGTTCAGCAACTCG	*Hind* III
	chsE1-U-F	CGGGATCCCCATCGGACGGCTCGAAGACG	*BamH* I
	chsE1-U-R	CCTCTAGACAGGATTCCCCTAGCTCGAG	*Xba* I
chsH2	chsH2-D-F	GCTCTAGATTCCCCGACAGTGAGGTCAG	*Xba* I
	chsH2-D-R	CCCAAGCTTGTGATCGGCTTGCCGTAG	*Hind* III
	chsH2-U-F	GAAGATCTCGCCGATATCCCGCTGCC	*Bgl* II
	chsH2-U-R	GCTCTAGAGGTCGGGCTGCTGCTGCC	*Xba* I
chsE5	chsE5-D-F	GCTCTAGAGGTGATGGTGCGGACCAG	*Xba* I
	chsE5-D-R	CCCAAGCTTCGAGTACGGCGGCCAGGC	*Hind* III
	chsE5-U-F	GAAGATCTCAGGGCACACCGGTCTCG	*Bgl* II
	chsE5-U-R	GCTCTAGAGGCAGCGCGACCGGCCAG	*Xba* I
ltp2	ltp2-D-F	GCTCTAGAACGGCGGGTACCGGGGTG	*Xba* I
	ltp2-D-R	CCAAGCTTGCGGCCACCGGTGCGAGC	*Hind* III
	ltp2-U-F	CGGGATCCAAGGACATCTTCGTCAACATCCTCACCG	*BamH* I
	ltp2-U-R	GCTCTAGAGGCGATCGCCGCTTTGCC	*Xba* I
ChsH3	chsH3-D-F	GCTCTAGAATCAGGCCTTTCCAGCGAGA	*Xba* I
	chsH3-D-R	CCCAAGCTTAGGGCGTGATCCTCGGTTC	*Hind* III
	chsH3-U-F	GAAGATCTGCCATCCGGACCCGTGTC	*Bgl* II
	chsH3-U-R	GCTCTAGAGCGCCGAGCCGTGACAAC	*Xba* I
echA19	echA19-D-	GCTCTAGAGATGAGGGTGTGTCCG	*Xba* I

	F	CG	
	echA19-D-R	CCCAAGCTTGTGCTGGCGAGCAGGAAC	*Hind* III
	echA19-U-F	GAAGATCTTCGAAATTCGGTGGCGAT	*Bgl* II
	echA19-U-R	GCTCTAGAGCCAAAGAGGGCCCGCGG	*Xba* I
chsE3	chsE3-D-F	GGAATTCGGCACCGAGCAGATCCTGCTCAC	*Xba* I
	chsE3-D-R	CCCAAGCTTTCGCTGGATTCCGACCCGTTCG	*Hind* III
	chsE3-U-F	GAAGATCTCGGCCGCCTCCGGGAGAGGTCG	*Bgl* II
	chsE3-U-R	GGAATTCGACCAGTTCACGGGCCGCGGCCTCAT	*Xba* I
casC	casC-D-F	GCTCTAGAACCGAGGTGCAGCTCAAC	*Xba* I
	casC-D-R	CCCAAGCTTTTCCCAGCGACCGTGCG	*Hind* III
	casC-U-F	GAAGATCTATATGGTTGCGCCGGAACA	*Bgl* II
	casC-U-R	GCTCTAGACACCAGTGACCGGACCGAA	*Xba* I

Table S2. NMR data of 3-OPD (Compound 17) in $CDCl_3$

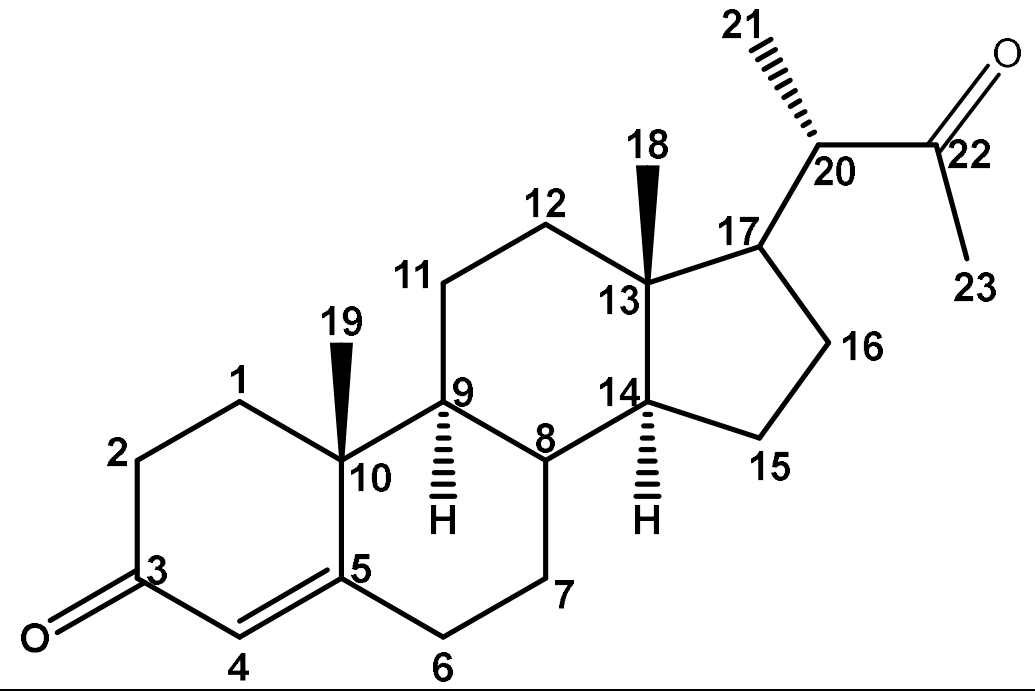

Carbon No.	^{13}C (δ, ppm)	^{1}H (δ, ppm)
1	34	2.3,2.26
2	32.89	2.47,2.38
3	199.61	-
4	123.9	5.74
5	171.34	-
6	31.98	1.83,1.03
7	28.11	2.11,1.73
8	35.6	1.54
9	52.04	1.6
10	35.72	-
11	21.02	1.57,1.41
12	38.61	1.32,1.28
13	42.68	-
14	55.36	1.08
15	24.44	1.66,1.19
16	39.54	2.01,1.97
17	53.75	0.96
18	12.27	0.76
19	17.41	1.2
20	50.32	2.52
21	16.4	1.14
22	212.76	-
23	27.42	2.13

a

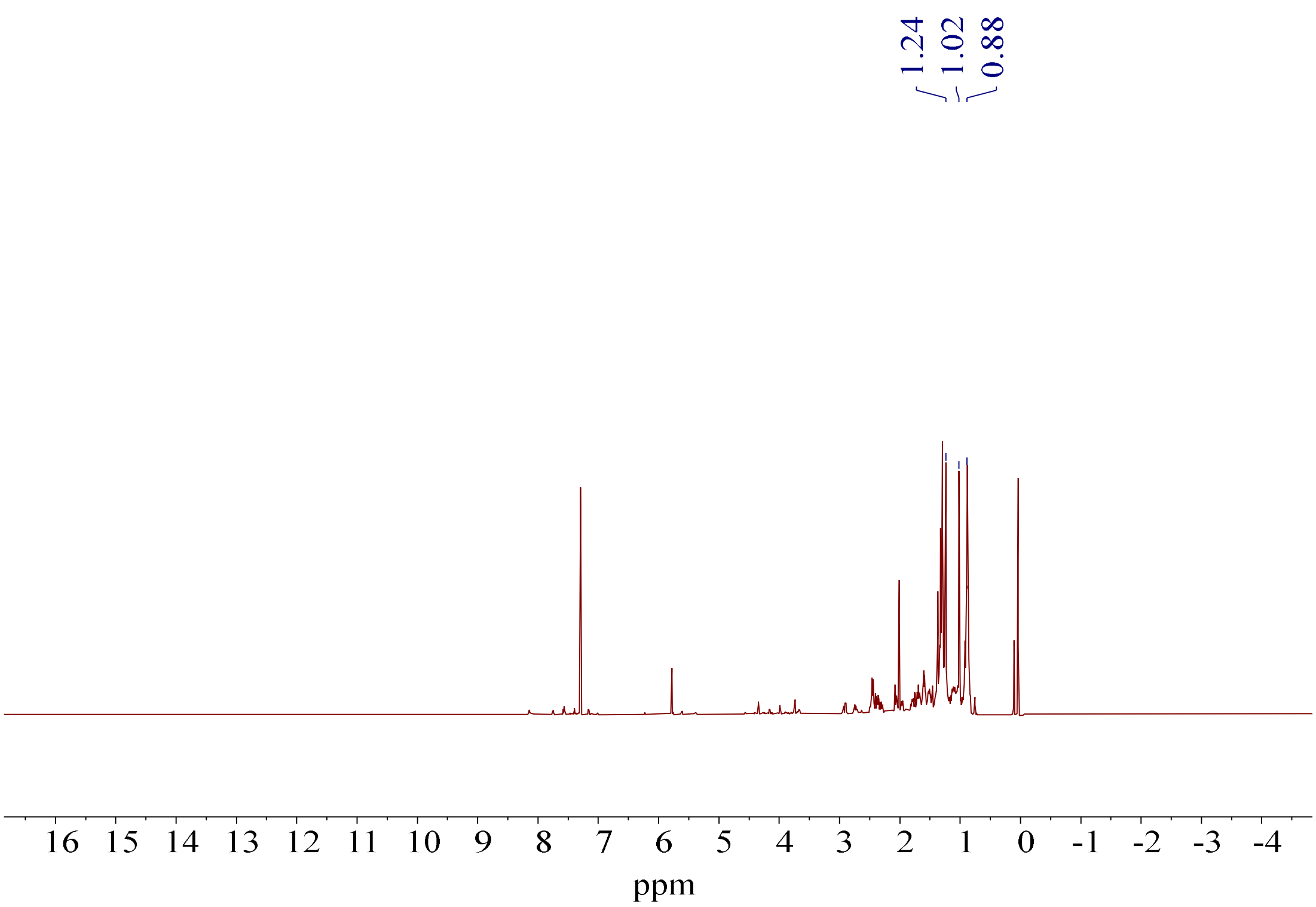

b

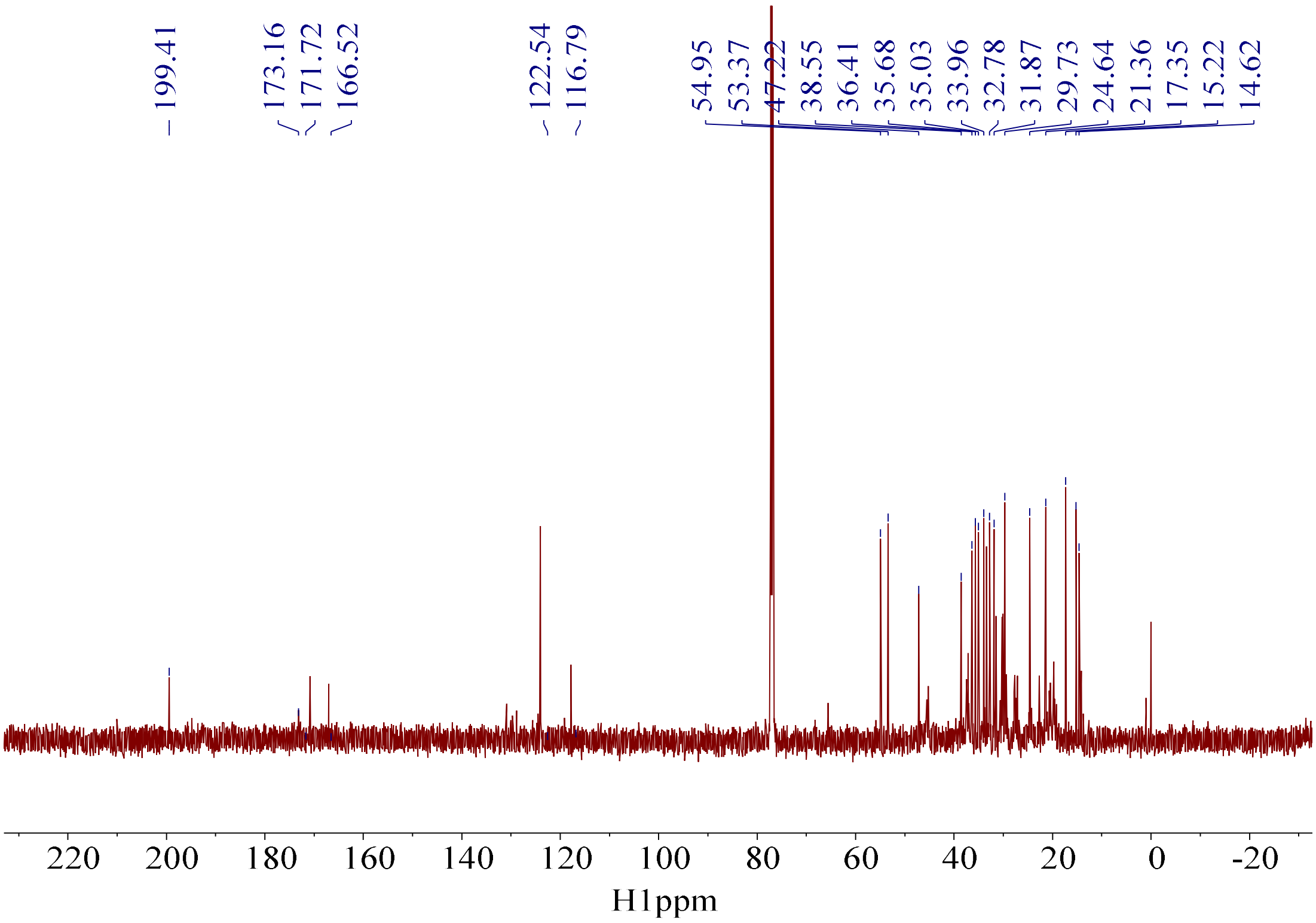

Fig S1. NMR spectra of 3-OPDC (Compound 13). a. ^{1}H NMR spectrum of 3-OPDC (Compound **12**); **b**. ^{13}C NMR spectrum of 3-OPDC (Compound **12**).

a

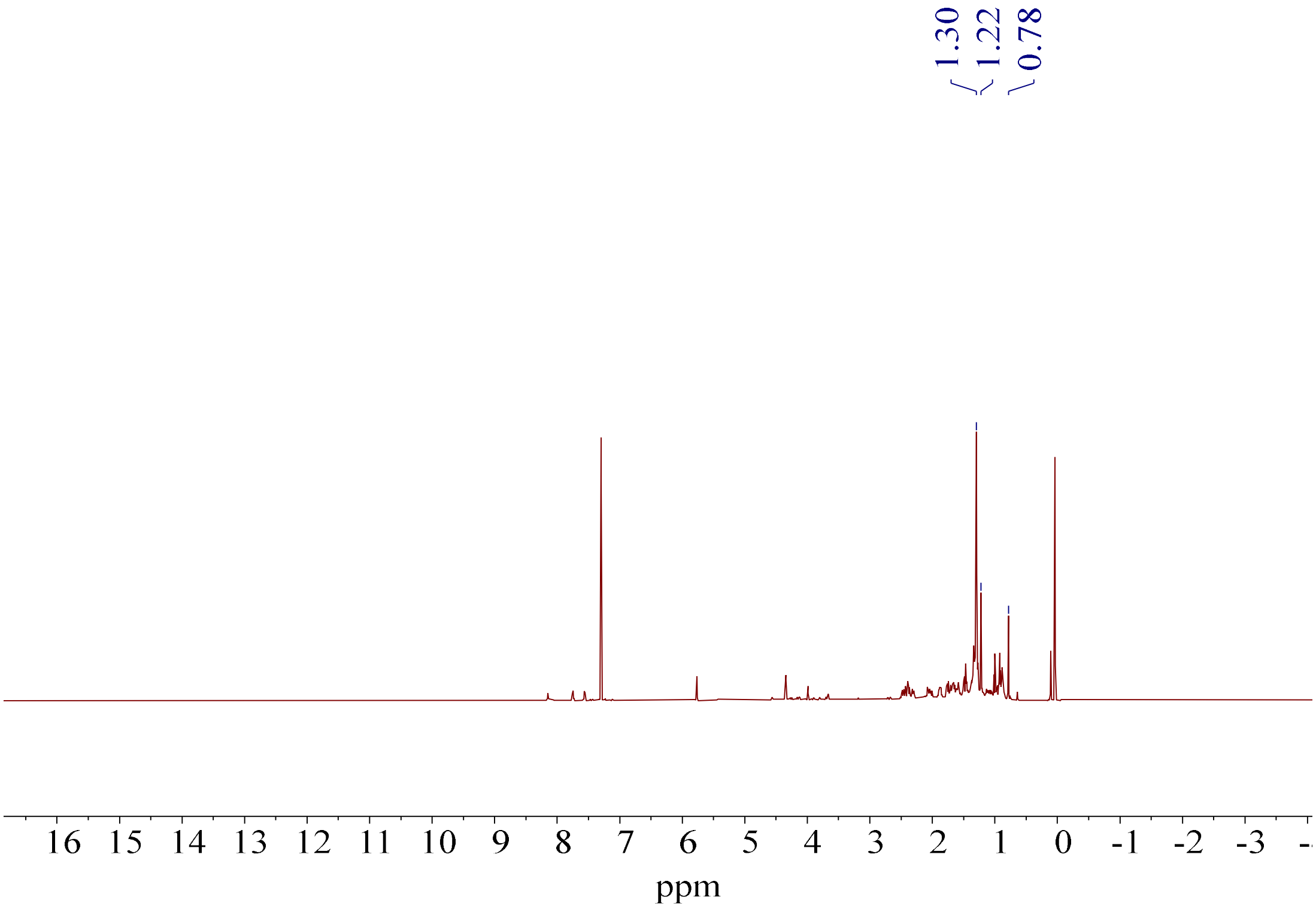
1.30
1.22
0.78
16 15 14 13 12 11 10 9 8 7 6 5 4 3 2 1 0 -1 -2 -3
ppm

b

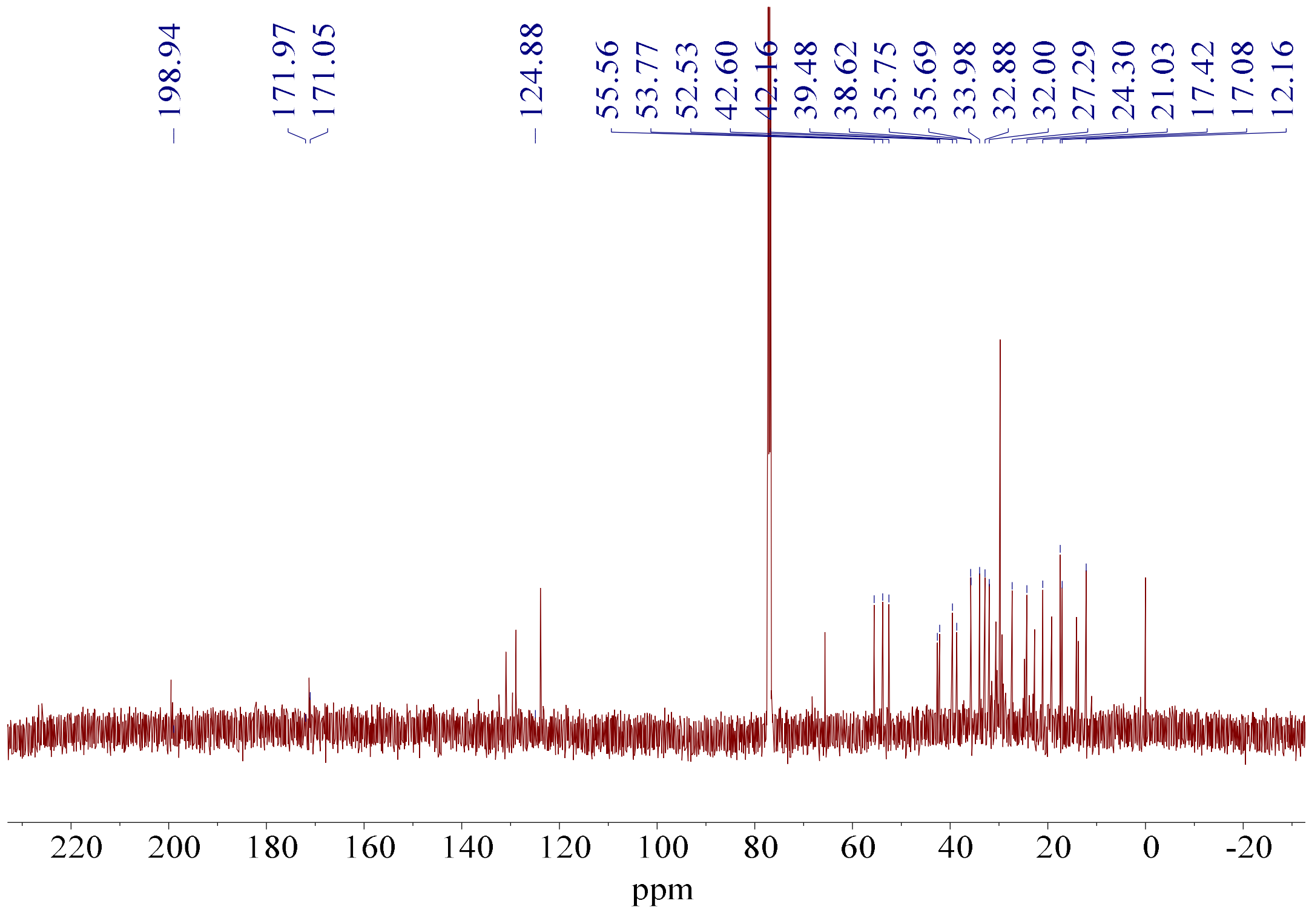
198.94
171.97
171.05
124.88
55.56
53.77
52.53
42.60
42.16
39.48
38.62
35.75
35.69
33.98
32.88
32.00
27.29
24.30
21.03
17.42
17.08
12.16
220 200 180 160 140 120 100 80 60 40 20 0 -20
ppm

c

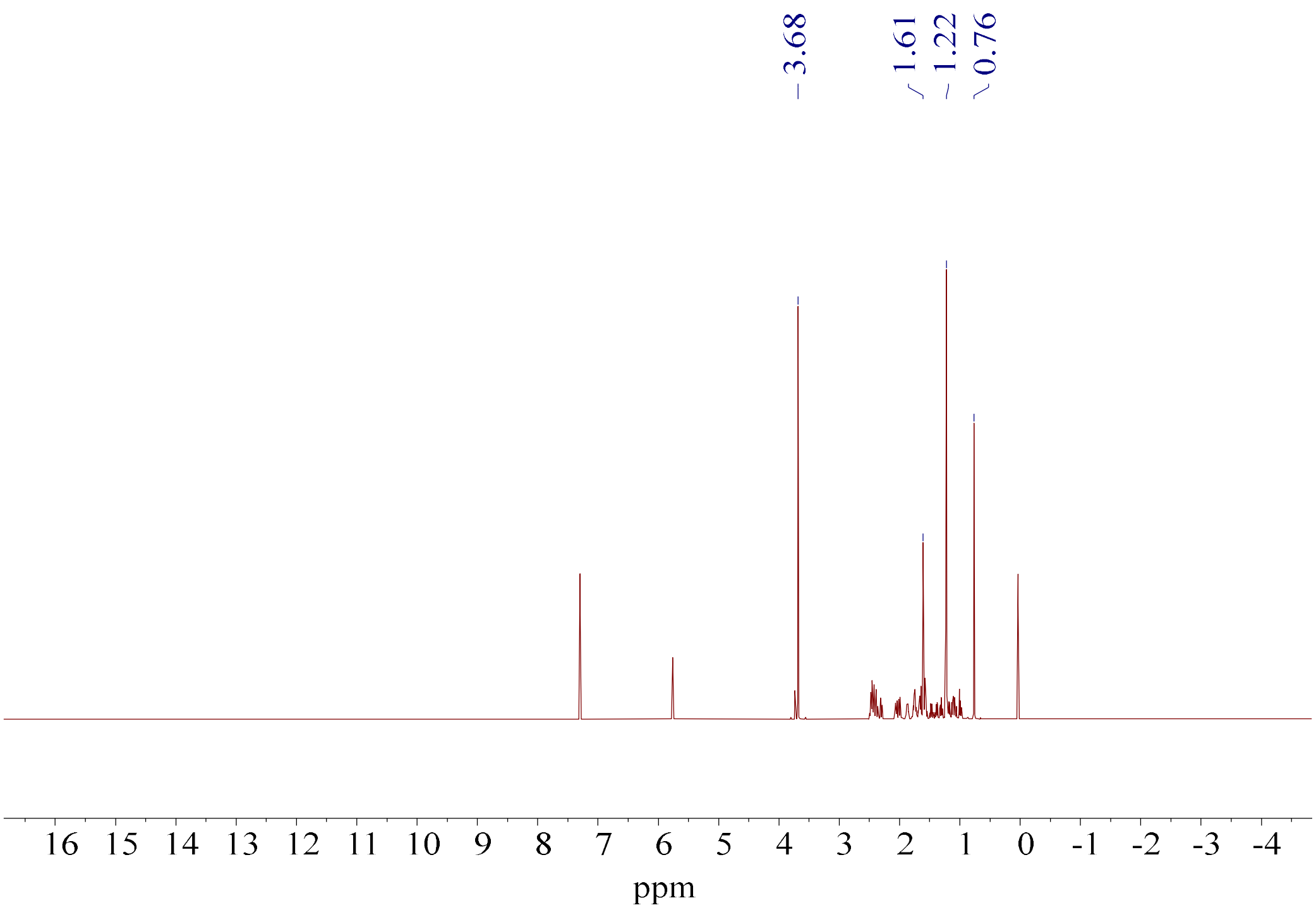
3.68
1.61
1.22
0.76
16 15 14 13 12 11 10 9 8 7 6 5 4 3 2 1 0 -1 -2 -3 -4
ppm

d

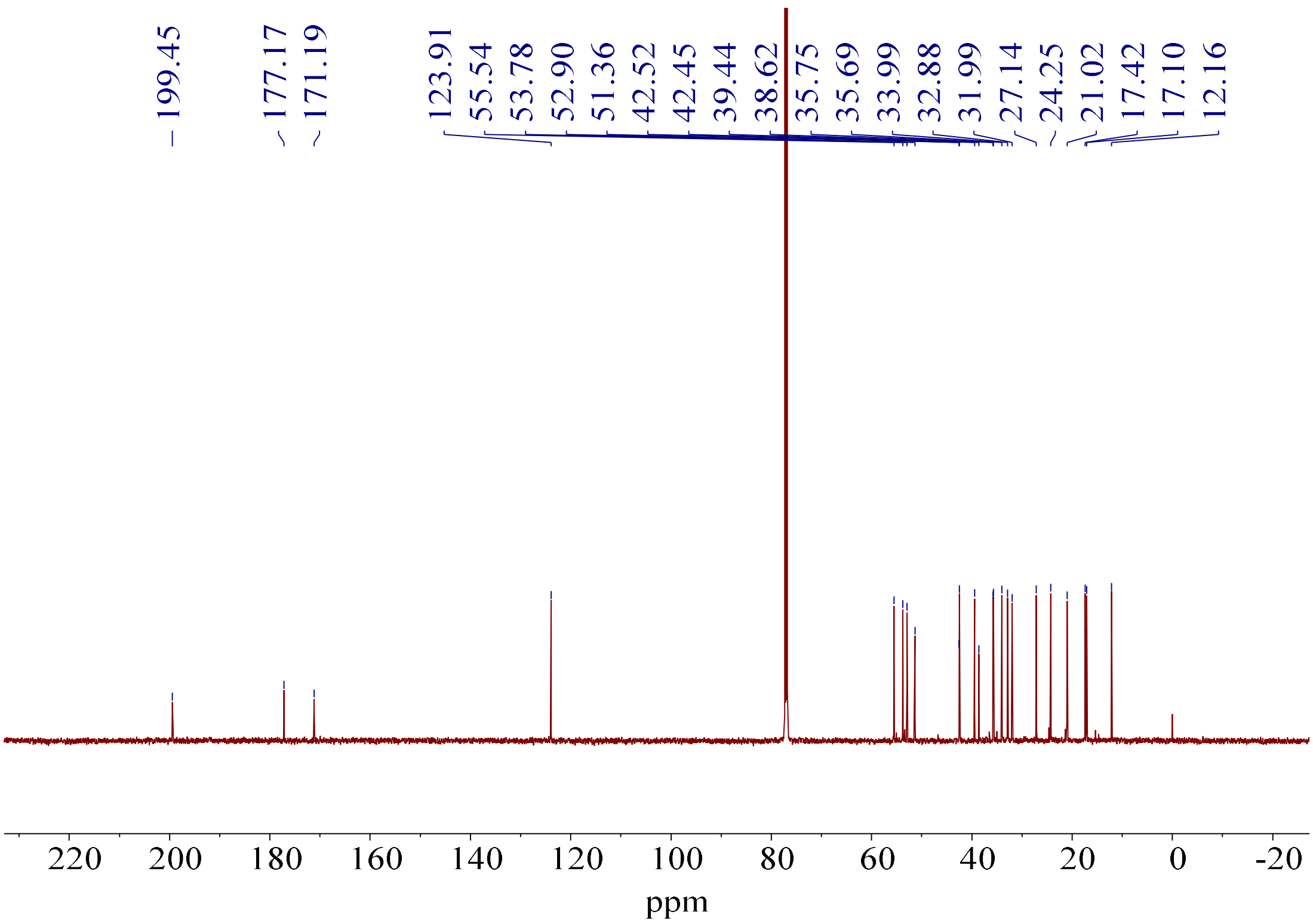
199.45
177.17
171.19
123.91
55.54
53.78
52.90
51.36
42.52
42.45
39.44
38.62
35.75
35.69
33.99
32.88
31.99
27.14
24.25
21.02
17.42
17.10
12.16
220 200 180 160 140 120 100 80 60 40 20 0 -20
ppm

e

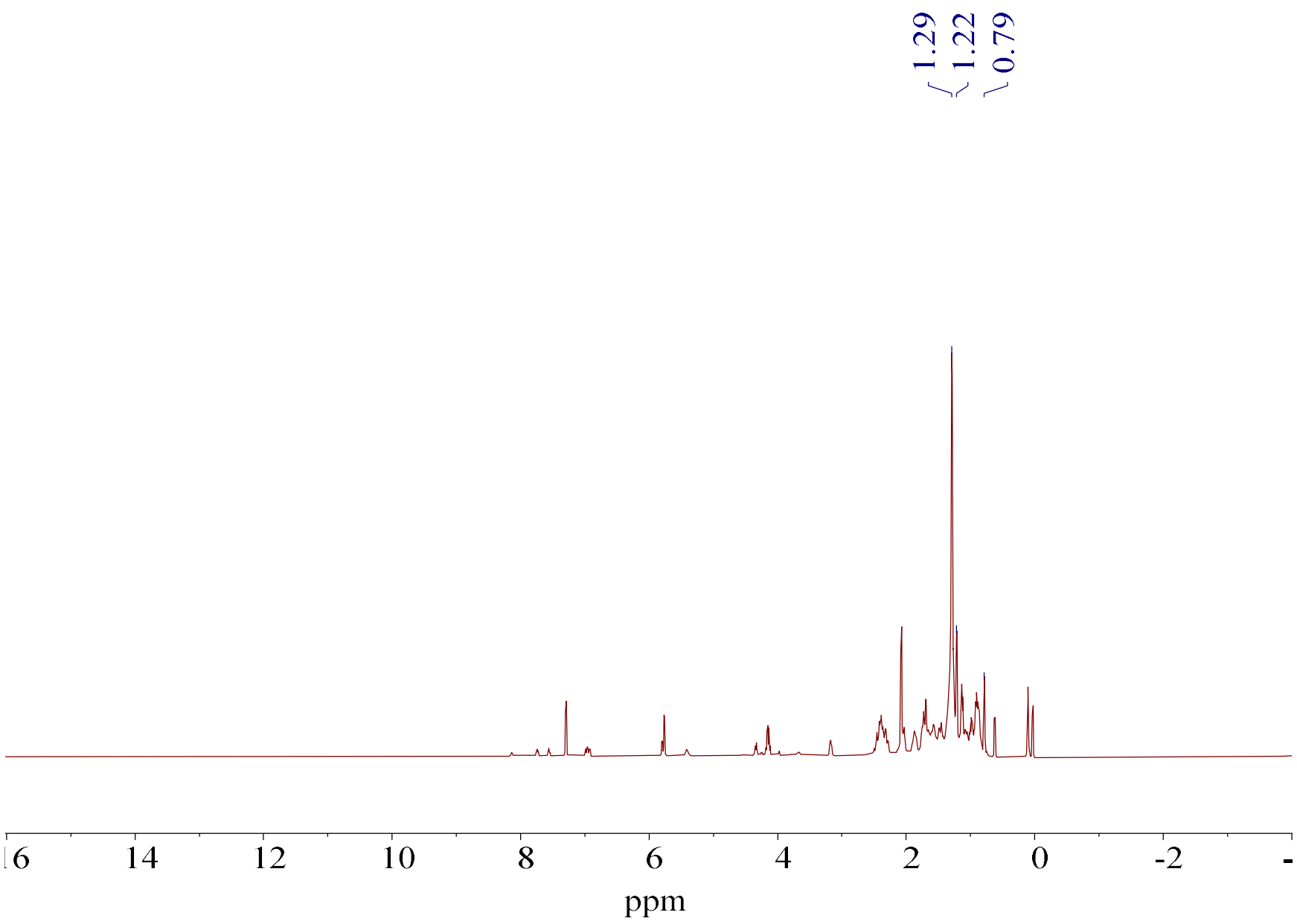

f

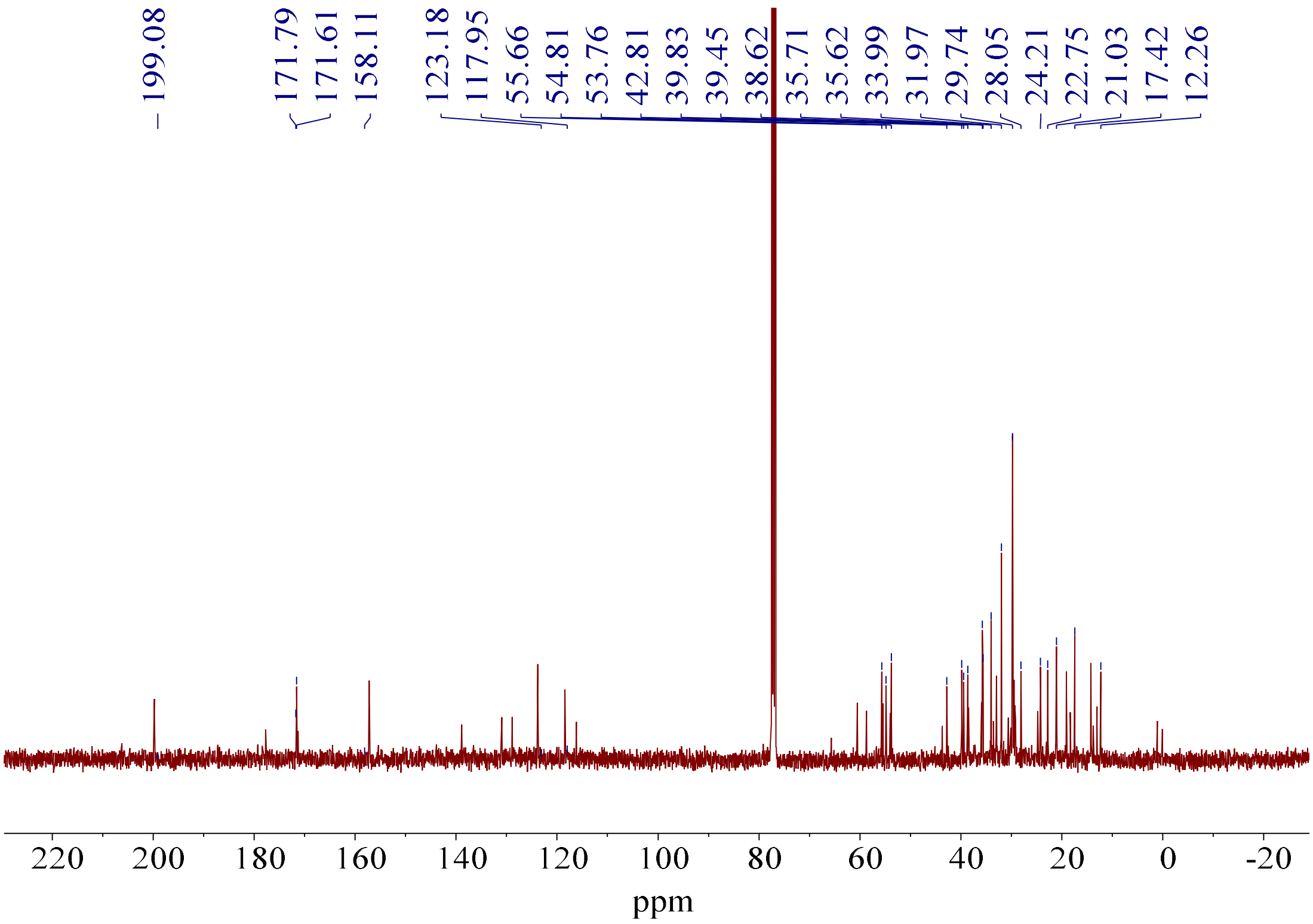

Fig S2. NMR spectra of 3-OPC (Compound 15), 3-OPCM (Compound 16), 3-OCDO (Compound 17). a. ^{1}H NMR spectrum of 3-OPC (Compound **15**); **b**. ^{13}C NMR of 3-OPC (Compound **16**). **c**. ^{1}H NMR spectrum of 3-OPCM (Compound **16**); **d**.^{13}C NMR spectrum of 3-OPCM (Compound **16**); **e**. ^{1}H NMR spectrum of 3-OCDO (Compound **17**); **f**. ^{13}C NMR spectrum of 3-OCDO (Compound **17**).

a

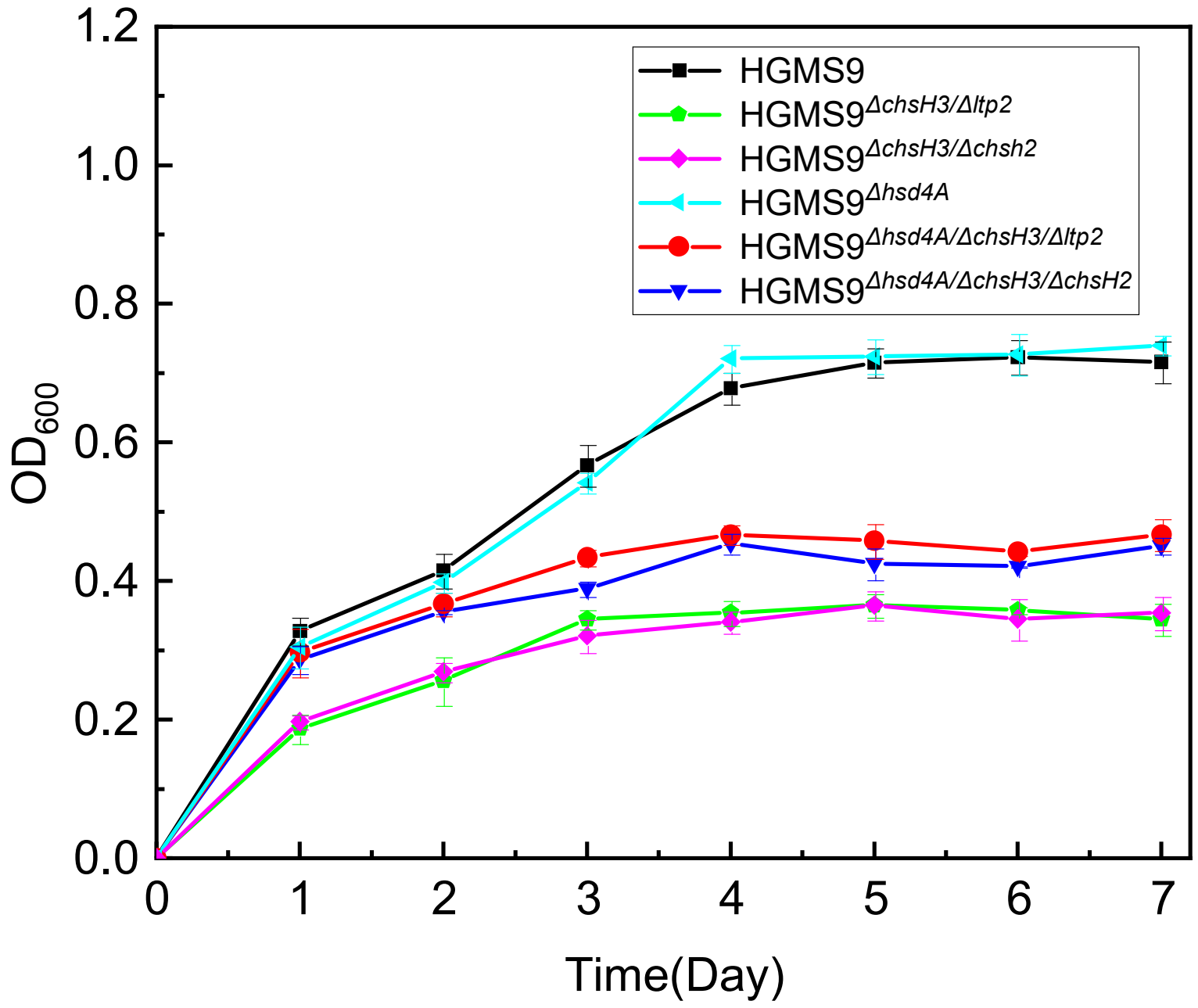

b

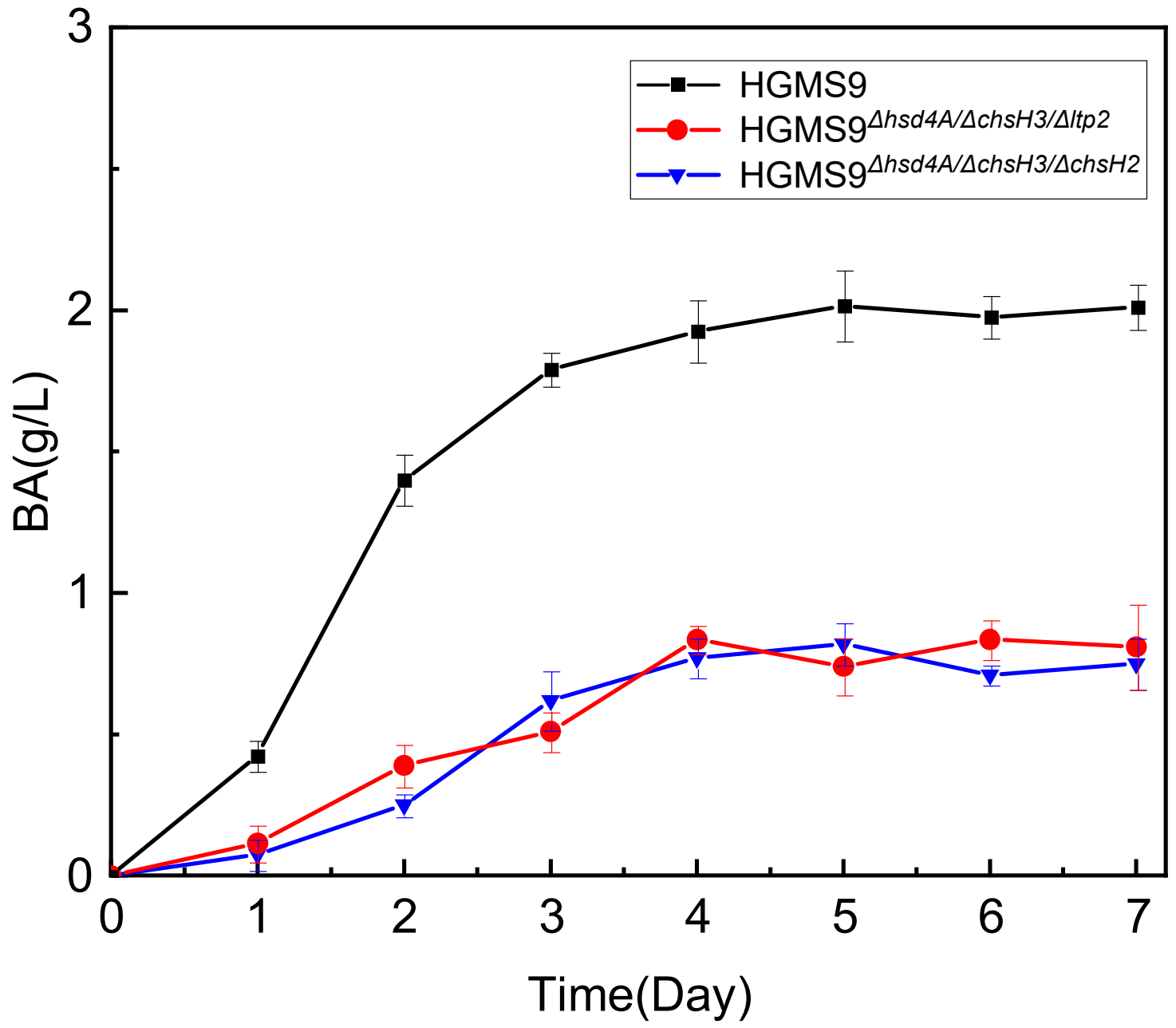

Fig S3. Comparison of cell growth and the BA yield of HGMS9, HGMS9$^{\Delta chsH3/\Delta ltp2}$, HGMS9$^{\Delta chsH3/\Delta chsH2}$, HGMS9$^{\Delta hsd4A}$, HGMS9$^{\Delta hsd4A/\Delta chsH3/ltp2}$ and HGMS9$^{\Delta hsd4A/\Delta chsH3/chsH2}$. a. The cell growth curves in the fermentation medium containing 5 g/L phytosterol. **b**. The bioconversion yield of BA (Compound **12**) with

the fermentation media 5 g/L of phytosterol.

a

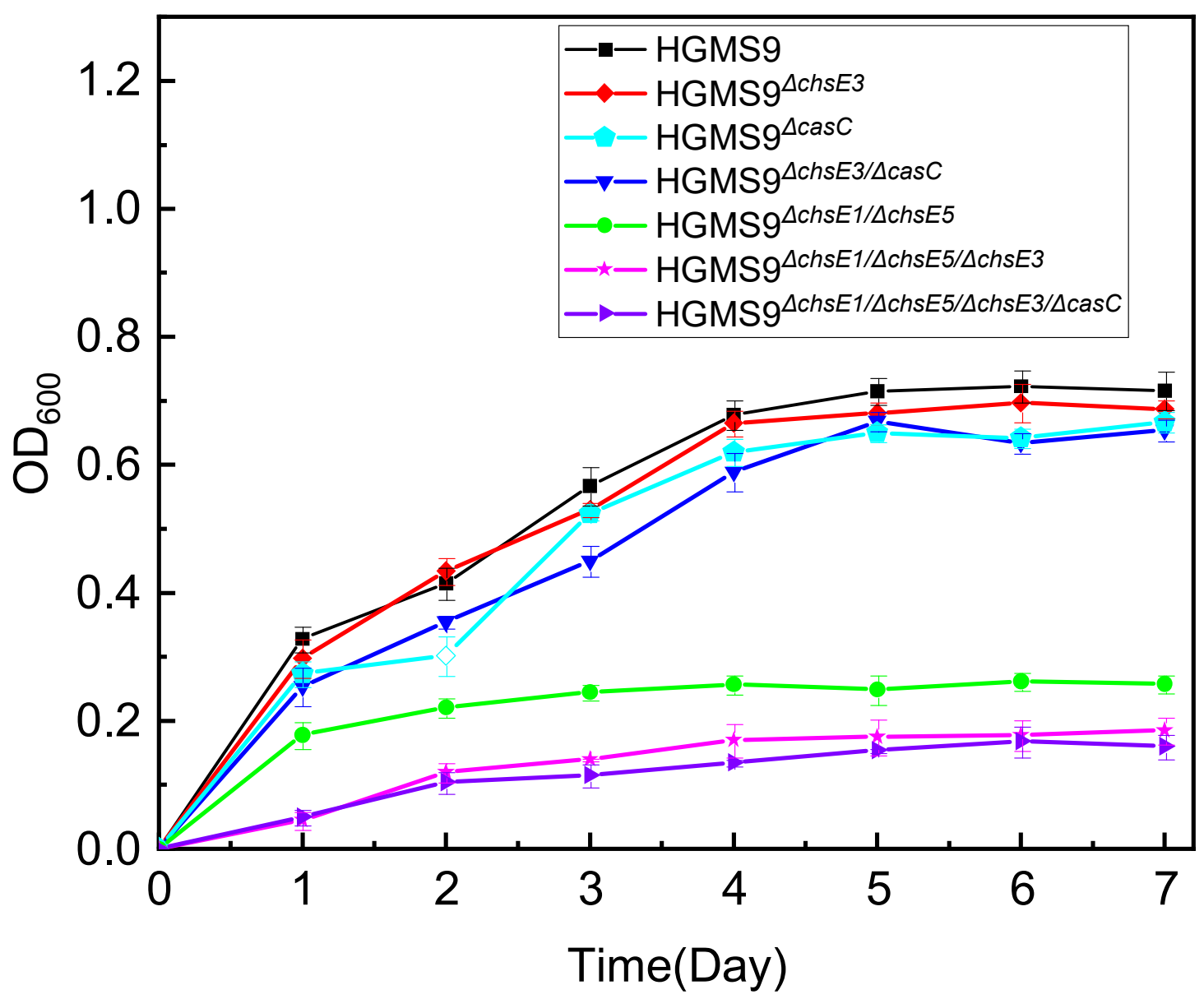

b

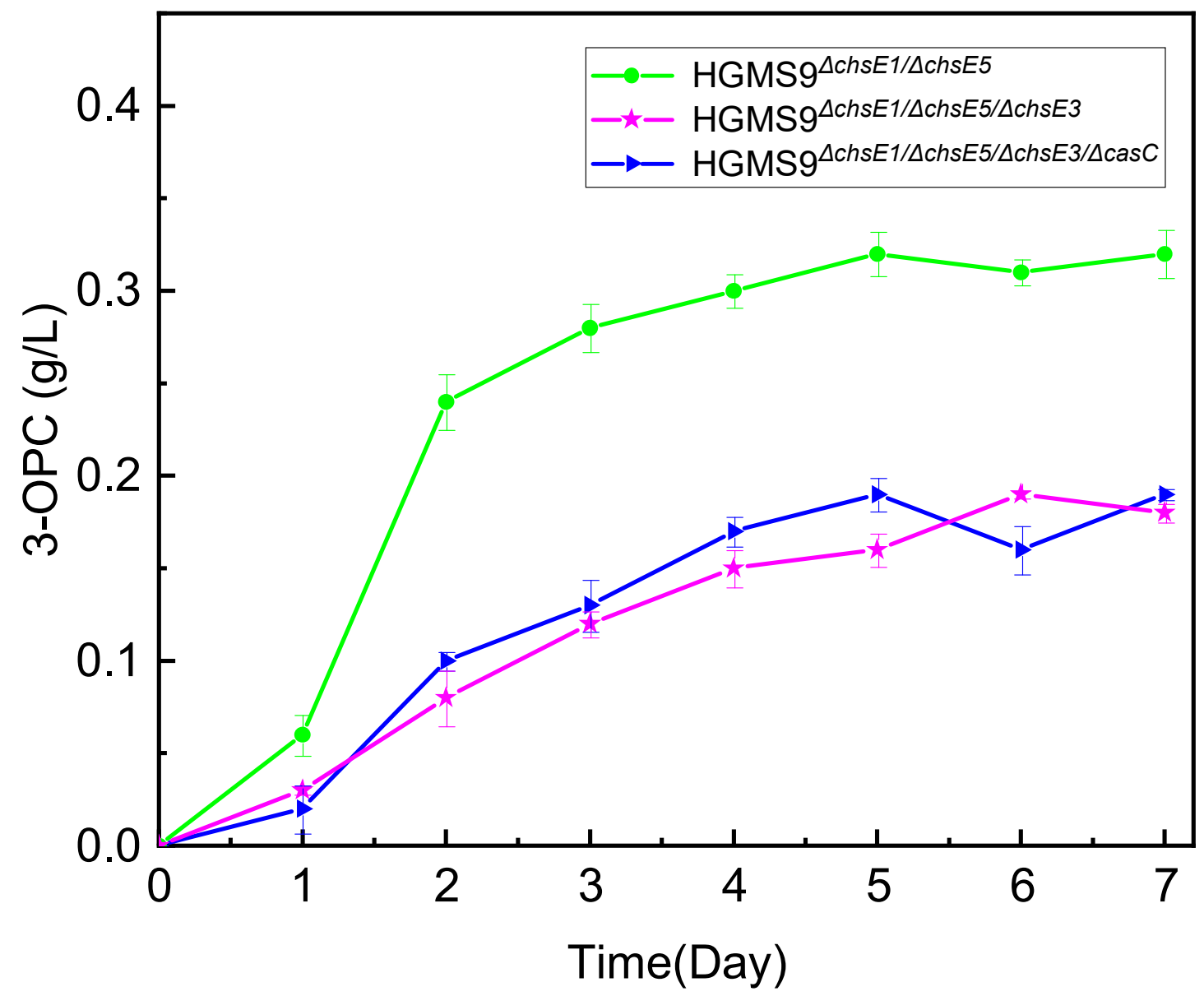

c

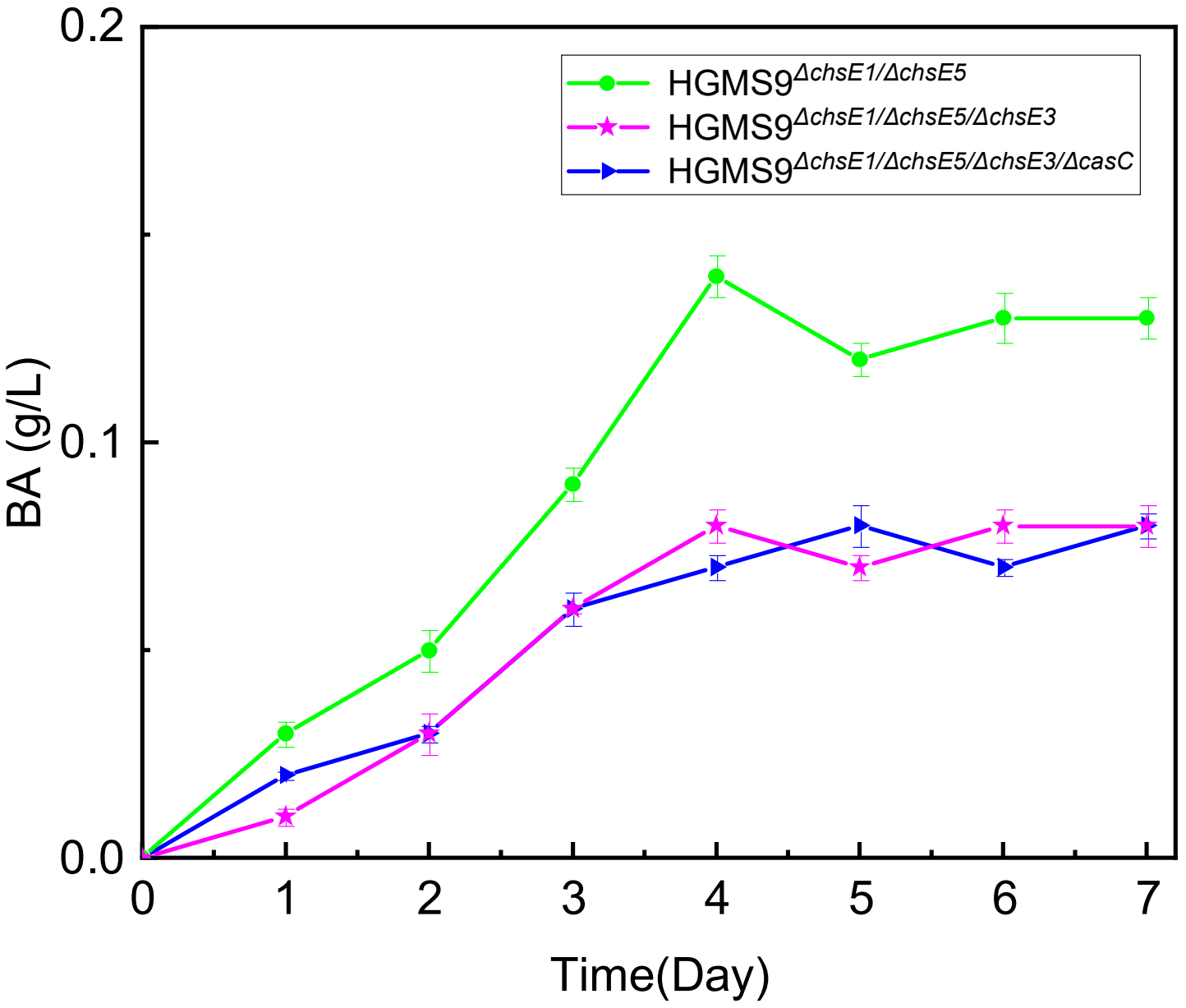

d

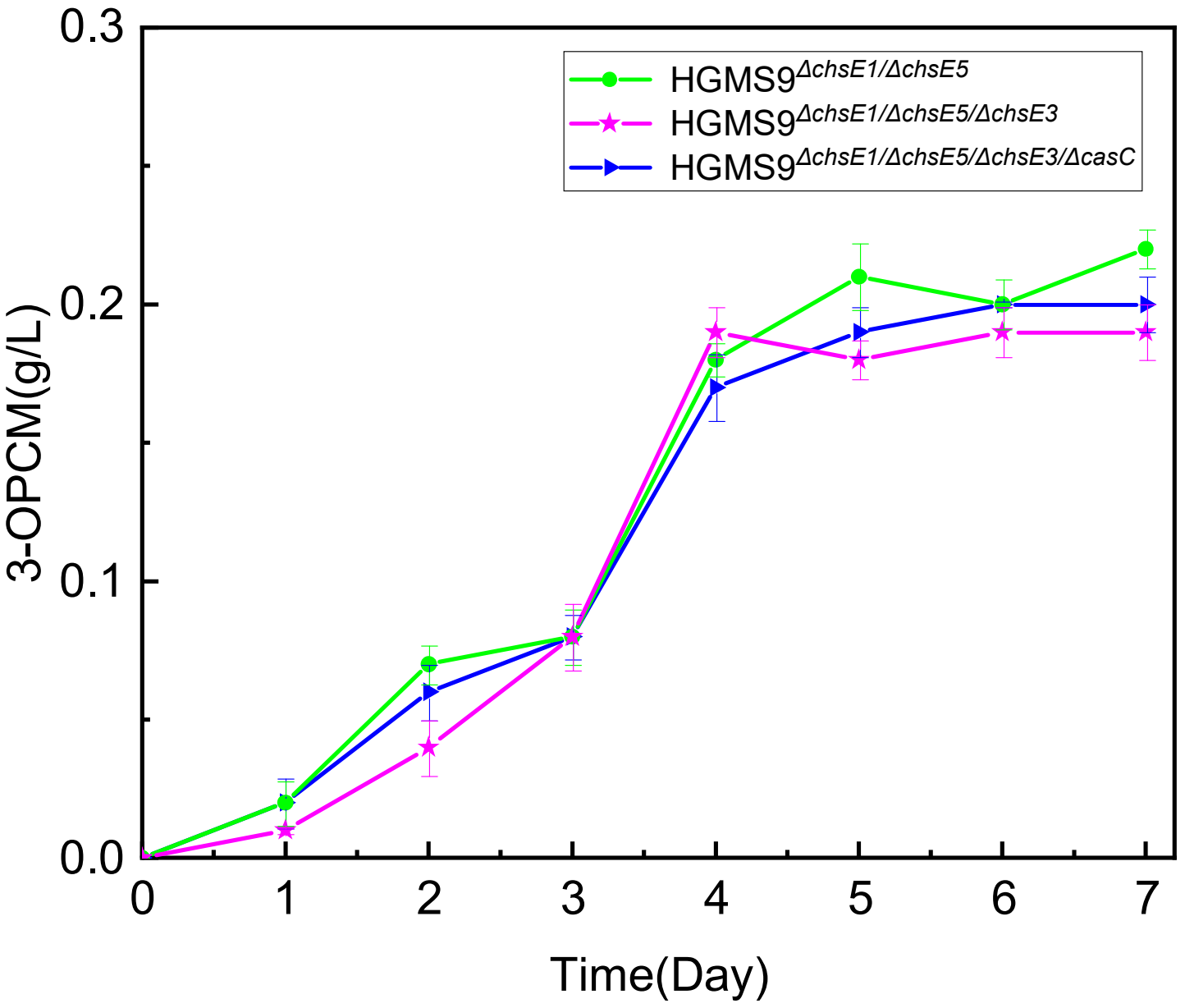

Figure S4. Comparison of cell growth and the intermediate yields of HGMS9, HGMS9$^{\Delta chsE3}$, HGMS9$^{\Delta casc}$, HGMS9$^{\Delta chsE3/\Delta casc}$, HGMS9$^{\Delta chsE1/\Delta chsE5}$, HGMS9$^{\Delta chsE1/\Delta chsE5/\Delta chsE3}$ and HGMS9$^{\Delta chsE1/\Delta chsE5/\Delta chsE3\Delta casc}$. a). The cell growth in the fermentation medium with 5 g/L phytosterol. **b)**. The yield of 3-OPC (Compound **15**) from 5 g/L phytosterol bioconversion. **c)**. The yield of BA from 5 g/L phytosterol bioconversion. **d)**. The yield of 3-OPCM (Compound **16**) from 5 g/L phytosterol bioconversion.

a

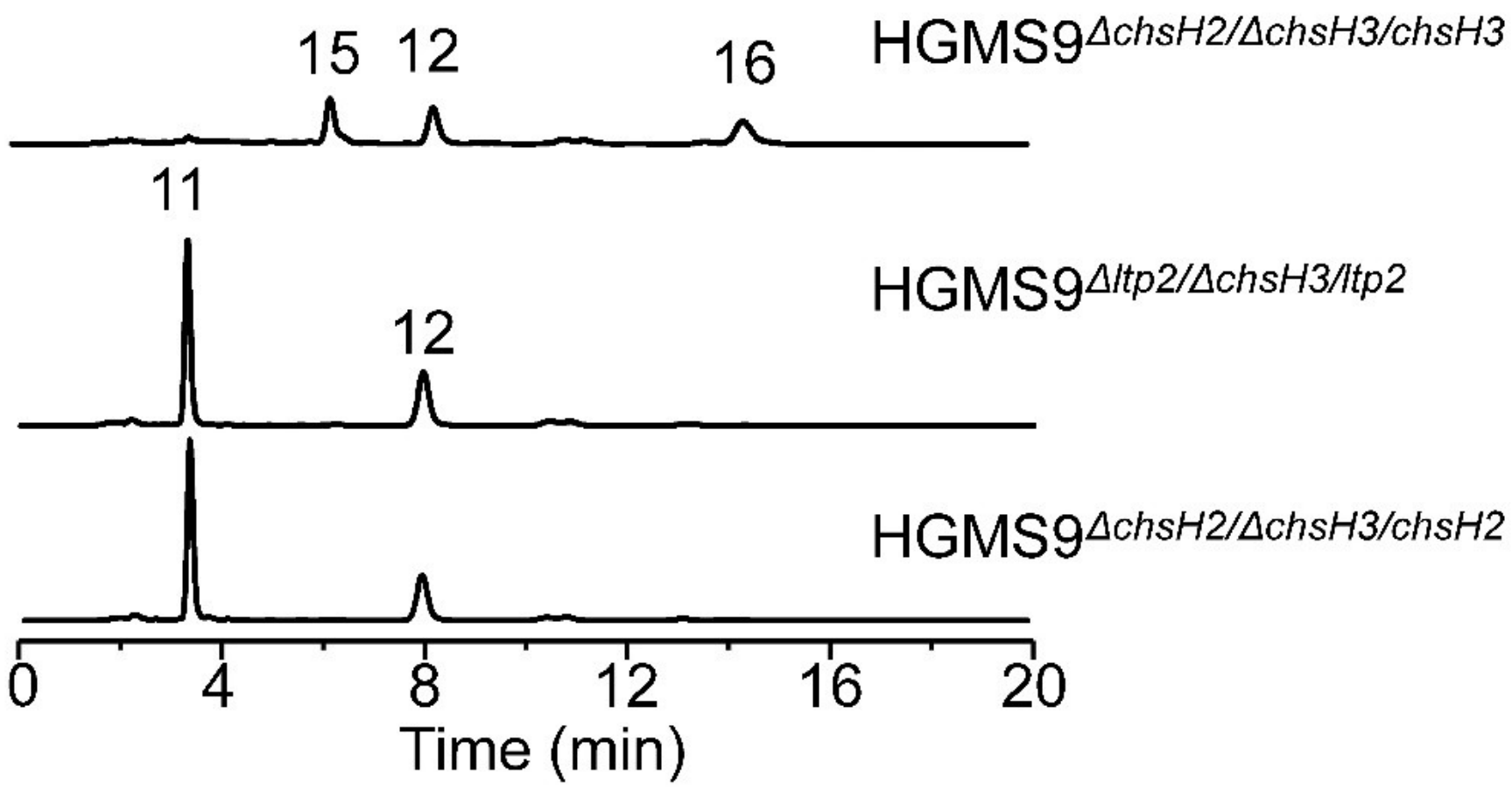
HGMS9^ΔchsH2/ΔchsH3/chsH3
15
12
16
11
HGMS9^Δltp2/ΔchsH3/ltp2
12
HGMS9^ΔchsH2/ΔchsH3/chsH2
0
4
8
12
16
20
Time (min)

b

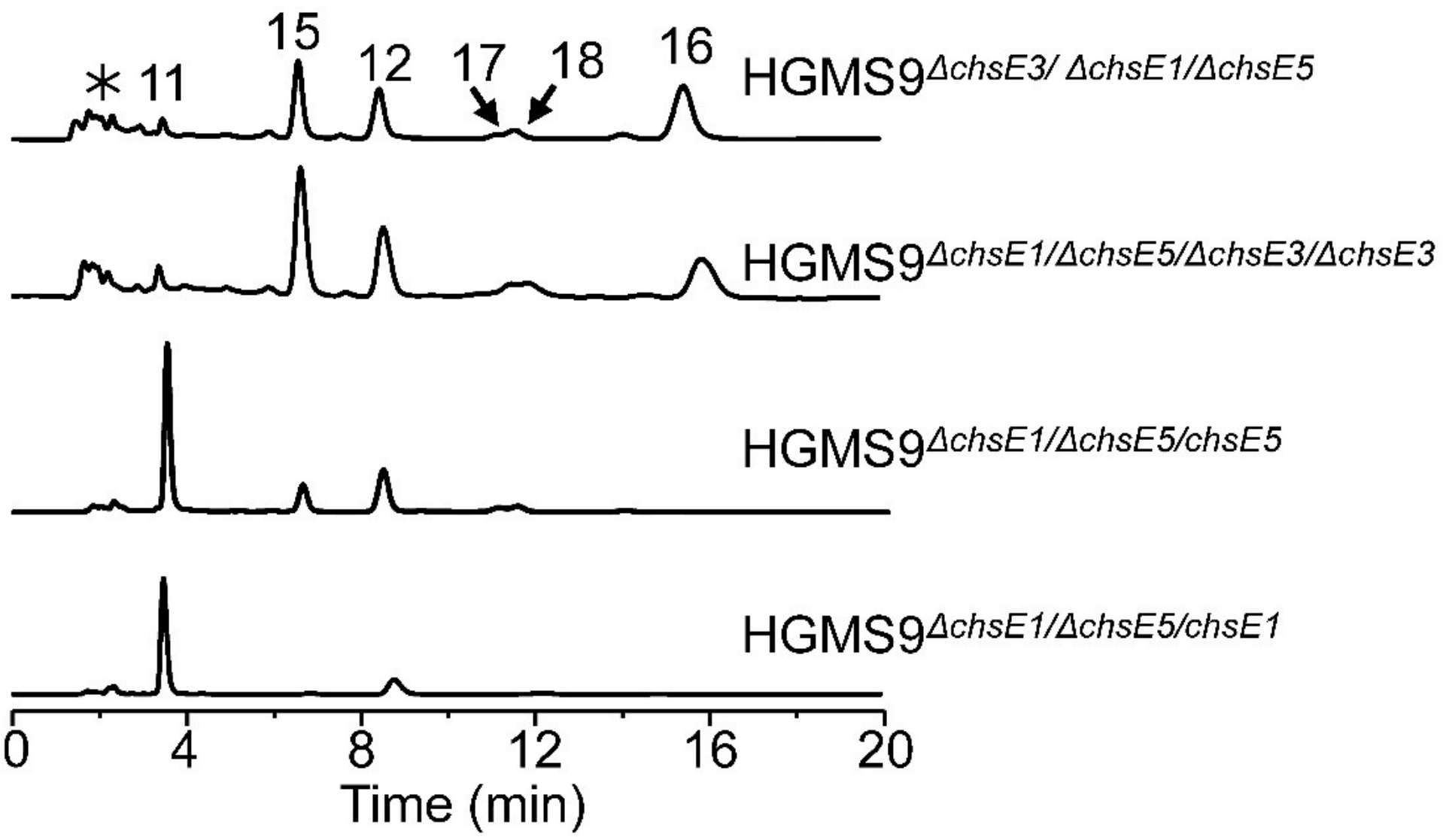
*
11
15
12
17
18
16
HGMS9^ΔchsE3/ ΔchsE1/ΔchsE5
HGMS9^ΔchsE1/ΔchsE5/ΔchsE3/ΔchsE3
HGMS9^ΔchsE1/ΔchsE5/chsE5
HGMS9^ΔchsE1/ΔchsE5/chsE1
0
4
8
12
16
20
Time (min)

c

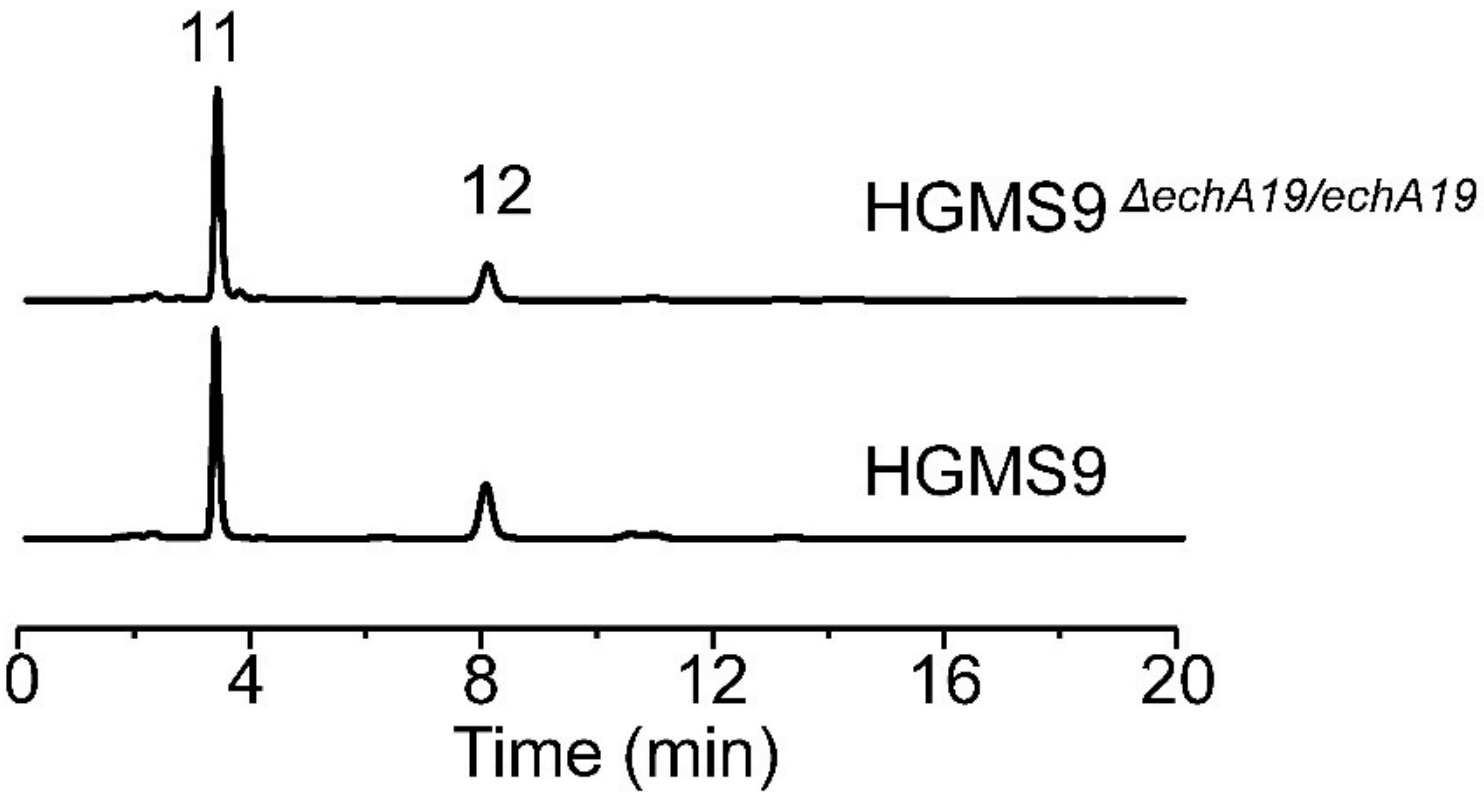

Fig S5. HPLC analysis of the fermentation broth extracts from the strains with gene complementation. **a**. Comparison of HGMS9$^{\Delta chsH2/\Delta chsH3/chsH2}$, HGMS9$^{\Delta ltp2/\Delta chsH3/ltp2}$ and HGMS9$^{\Delta chsH2/\Delta chsH3/chsH3}$. **b**. Comparison of HGMS9$^{\Delta chsE1/\Delta chsE5/chsE1}$, HGMS9$^{\Delta chsE1/\Delta chsE5/chsE5}$, HGMS9$^{\Delta chsE1/\Delta chsE5/\Delta chsE3/chsE3}$ and HGMS9$^{\Delta chsE3/\Delta chsE1/\Delta chsE5}$. **c**. Comparison of HGMS9 and HGMS9$^{\Delta echA19/echA19}$
